# Archetype-based stratification to inform sub-national tailoring of malaria interventions in Zambia

**DOI:** 10.1186/s12936-026-05998-7

**Published:** 2026-06-18

**Authors:** Chilochibi Chiziba, Japhet Chiwaula, Busiku Hamainza, Sampa Chitambala-Otiono, Sheetal Silal

**Affiliations:** 1 Modelling and Simulation Hub, Africa, Department of Statistical Sciences, University of Cape Town, Cape Town, South Africa; 2 National Malaria Elimination Centre, Ministry of Health, Lusaka, Zambia; 3 Programme Coordination Unit, Ministry of Health, Lusaka, Zambia; 4 Nuffield Department of Medicine, Centre for Global Health, Oxford University, Oxford, UK

**Keywords:** Malaria, Archetype, Mapping, Cluster, Health facility catchment area, Zambia

## Abstract

**Introduction:**

Although Zambia does not rank among the highest contributors to global malaria cases, in 2023, it had approximately eleven million cases. Furthermore, Zambia’s malaria incidence distribution is spatially heterogeneous at fine scales such as health facility catchment areas (HFCAs). HFCA-level malaria incidence stratification currently guides implementation and targeting approaches. We aim to enhance the current stratification approach by incorporating archetypical characteristics associated with malaria, including environmental, access, epidemiological, and socioeconomic factors. An archetype-based stratification approach may inform the tailoring of interventions to accelerate progress toward elimination.

**Methods:**

We used literature-informed data from 2017 to 2024 from the Zambia Health Information Management System and publicly available datasets. A zero-inflated negative binomial mixed-effects regression model was fitted to identify significant variables associated with malaria heterogeneity at the HFCA level. Significant variables were used to archetype HFCAs using Clustering Large Applications algorithm.

**Results:**

Significant variables included: walking travel time to nearest health facility; percentage of malaria attributed to pregnant women; percentage of malaria attributed to children under five (U5); Children U5’s bednet (Long-lasting insecticide-treated nets or Insecticide-treated nets) coverage; bednet use rate; elevation; enhanced vegetation index; precipitation; and housing quality. Four new archetypes were derived. Relative to malaria incidence-based stratification, the archetype-based stratification exhibited spatiotemporal stability over time. Across all four new archetypes, elevation, bednet use rate, and housing quality emerged as the top-contributing features. However, all four archetypes exhibited distinct features defining their identity.

**Conclusions:**

The study proposes embedding archetypical characteristics in the malaria incidence-based stratification approach to better inform intervention tailoring and prioritisation based on the distinct archetype features associated with malaria at the HFCAs level. Furthermore, the study provides a precursor for simulating potential intervention mixes based on malaria transmission archetypical characteristics, which can provide decision-makers with projected impact of varied investments in HFCAs with similar archetypical features.

**Supplementary Information:**

The online version contains supplementary material available at https://doi.org/10.1186/s12936-026-05998-7.

## Introduction

Similar to many infectious diseases, malaria is characterised by clustering tendencies, perpetuated by the heterogeneity in various factors that provide a suitable environment for mosquitoes to thrive and parasite transmission [1]. The clustering trend is observed at the global level, where more than 90% of the more than 200 million global malaria cases have been historically attributed to sub-Saharan Africa [2, 3]. The clustering tendency is also observed at national scales, including Zambia [1]. Like many sub-Saharan countries, Zambia is also burdened with malaria, with approximately eleven million cases reported at health facilities and community structures, and contributing about 1.4% to the global malaria burden as of 2023 [2, 4, 5]. Despite Zambia not being among the highest contributors to global malaria cases, its malaria risk distribution (annual cases per 1,000 population) and malaria parasite prevalence are characterised by spatial heterogeneity over smaller spatial boundaries, such as health facility catchment areas (HFCAs) [6].

The underlying spatial differences in the malaria burden in Zambia are driven by variations in behavioural, access, environmental, ecological, socioeconomic, demographic, and malaria intervention histories [6–8]. Furthermore, the Zambia National Malaria Elimination Centre (NMEC) identified rural populations, people living at lower altitudes and near water bodies, individuals with low socioeconomic status, mobile populations, pregnant women, and children under five (U5) as vulnerable populations for malaria infection [6].

Over the years, the government of Zambia and several partnering organisations have delivered various malaria intervention strategies [6, 9]. Building on past experiences and the World Health Organisation’s (WHO) recommendations, Zambia’s 2022–2026 National Malaria Elimination Strategic Plan (NMESP) prescribed intervention packages for HFCAs, based on their malaria incidence profiles, which is a stratification of annual cases per 1,000 population [6, 9–11]. For example, HFCAs with greater than 500 annual cases per 1,000 people pursue intensified control strategies (reducing transmission), while HFCAs with fewer than 50 cases pursue pre-elimination strategies (interrupting local transmission) [6]. However, several interventions function as control strategies or pre-elimination strategies [6, 12]. Considering the widening shortfalls in funding for malaria, not all interventions per strategy can be deployed [11, 13]. As such, it is important to determine which intervention to implement and where to gain the most from these interventions [8, 14, 15].

In the call for optimal allocation of interventions, the WHO emphasises doing away with a one-size-fits-all approach and rather applying interventions that are tailored to local settings [12]. This is because the effectiveness of interventions has been closely linked to how well they align with the unique contextual realities of specific areas [1, 8, 16, 17]. To align with the global emphasis of deploying optimal interventions and to contribute towards the global malaria goals, the Zambian government and partnering organisations have been taking deliberate actions to pilot and deploy targeted interventions in selected parts of the country [1, 6, 18]. However, other than the HFCA malaria incidence profiling, no country-wide stratification has been conducted to inform the tailoring of interventions at a national level based on malaria-related drivers or factors that may be linked to intervention effectiveness [6].

The tailoring of interventions beyond malaria incidence profiles requires incorporating factors that drive transmission [17]. The incorporation of malaria drivers in the stratification process not only provides a relatively robust framework for intervention tailoring but also identifies opportunities for de-prioritisation and reallocation of resources [19]. Due to the clustering tendency of malaria, several studies, including Ozodiegwu et al. [15], have used cluster analysis to generate country archetypical malaria profiles using malaria-related drivers as part of their stratification process.

To enhance Zambia’s intervention targeting approaches, this study leverages and builds on the existing stratification strategy, which allocates interventions based on HFCA-level malaria incidence profiles. The study achieves this by identifying key drivers associated with malaria at the HFCA level and uses them to cluster HFCAs into new archetypes with similar characteristics. It then integrates the archetypes with the malaria incidence levels prescribed by the NMSP. Furthermore, the study examines the spatio-temporal stability of the archetypes compared to the malaria incidence based HFCA stratification. To inform intervention tailoring, the study also highlights the features mostly contributing to the clustering tendency per individual archetype.

## Methods

### Data

Yearly HFCA level data from 2017 to 2024, accessed on 13/04/2025 from the Zambia Health Analytics Platform (ZHAP), along with publicly available geospatial data, were used in this study. The platform data are originally reported as aggregates at the HFCA administrative level in the national Health Management Information Systems; therefore, it is fully anonymised. Furthermore, this study was reviewed and approved by two ethics committees as part of three sub-studies under one main study. Geospatial variables such as walking time to health facilities (Additional File 1 provides a comprehensive list, descriptions, and sources of all variables explored) were extracted from raster files using the terra package [20] using the HFCA boundaries shapefile. The HFCA boundaries shapefile was obtained from the NMEC and was generated in 2023. To maintain the geospatial capability of the analysis, the HFCA boundaries were used as the master source for merging HFCA data. Of the 3,285 unique HFCAs identified in ZHAP from 2017 to 2024, 382 did not report malaria incidence over the analysis period and were therefore excluded from the analysis dataset. Among the remaining 2,903 HFCAs, 335 (11.5%) were further excluded because they did not have a matching HFCA shapefile boundary; the reasons for this included health posts nested within a parent HFCA, recently split facilities, and referral hospitals without formal catchment areas. The remaining 2,568 HFCAs were retained for the subsequent analyses. A descriptive comparison of the excluded and retained HFCAs, including their urban/rural composition, is presented in Additional File 6.

### Descriptive analysis

To understand the distribution of malaria burden at the HFCA level, we categorised annual malaria cases using the NMEC stratification: ‘no malaria’ (0 cases), ‘very low’ (1–49), ‘low’ (50–199), ‘moderate’ (200–499), and ‘high’ (> 500) cases per 1,000 population per year. However, we grouped “no malaria” (0 cases) and “very low” (between 1 and 49 cases per 1,000 population per year) into a single category labelled “very low” (0–49) because there were very few “no malaria” HFCAs [6]. These categories were then mapped to visualise changes in malaria incidence stratification across HFCAs nationwide from 2017 to 2024, along with a trend bar chart.

### Covariate selection for cluster analysis

To select variables for cluster analysis, the study needed to reduce the number of variables to the ones associated with malaria incidence at the HFCA level. As such, available literature-informed malaria-related drivers and intervention data in Additional File 1 were examined for potential inclusion in the cluster analysis. The study explored three variable reduction methods, including Principal Component Analysis (PCA), Singular Value Decomposition (SVD), and regression. The regression method consisted of two steps: a Pearson correlation coefficient test to identify and eliminate correlated variables, and a zero-inflated negative binomial mixed-effects regression model to determine which variables were significantly associated with malaria incidence. From the Pearson correlation coefficient test, variables that were not correlated with others were considered for the regression. For pairs of variables with a correlation coefficient greater than 0.6, only the variable with the stronger association with malaria was retained, while the other was excluded [21]. We used the 0.6 threshold rather than the more conventional 0.7 because the candidate variable set explored in Additional File 1 contains composite indices whose components are themselves candidate variables such as wealth index (which incorporates housing quality) and relative humidity (which is a function of rainfall and temperature). A relatively strict threshold of 0.6 avoided the redundant inclusion of these composite indices alongside their component variables. Furthermore, the Pearson correlation coefficient test approach prevented the subsequent statistical model from multicollinearity and informed the elimination of relatively unimportant variables [21].

A zero-inflated negative binomial mixed effects regression model, with the annual number of malaria cases in the HFCA as the dependent variable and the covariates identified in the Pearson correlation coefficient test process as regressors, was fit [22]. The model was fit using the glmmTMB package in R and included the HFCA unique identification number as a random effect nested at the province level [22]. The province nesting was included to account for varying provincial dynamics, such as political and donor presence. Furthermore, variables with *p* < 0.05 were considered statistically significant and retained for the subsequent cluster analysis. Full model fitting and variable transformation procedures are outlined in Additional file 1’s “Variable transformation and regression procedure” section.

### Clustering approach

The HFCA level PCA and SVD reduced, and significant covariates in the regression model were each used to classify HFCAs. The study explored hierarchical clustering, K-means, K-Medoids Clustering (PAM), and Clustering Large Applications (CLARA) algorithms using data from the three data reduction methods. The process involved scaling the variables to have a mean of zero and a standard deviation of one, using the scale function from the base package in R. Thereafter, the optimal number of clusters was determined for each variable reduction methods and clustering algorithm using the Ward’s method for hierarchical clustering and the silhouette and within-cluster sum of square (wss) methods for K-means, PAM, and CLARA algorithms. To select the appropriate data reduction method, clustering algorithm, and the optimal number of clusters, the study evaluated the clustering quality for each combination. Clustering quality was evaluated using the Silhouette Score displayed in Eq. 1 [23–25], which quantifies how well each cluster point fits within its assigned cluster compared to its proximity to points in other clusters. Having a scale ranging from −1 to 1, a higher Silhouette Score indicates that clusters are clearly distinguished and apart [23–25].

1 $$Silhoutte\;score\left( i \right) = \frac{{b_{i} - a_{i} }}{{\max \left( {b_{i} ,a_{i} } \right)}}$$

where,

- ${a}_{i}$ is the smallest mean distance of i to points in the cluster to which it was assigned [23, 24].
- ${b}_{i}$ is the smallest mean distance of i to the points in the nearest cluster to which it was not assigned [23, 24].

After selecting the clustering algorithm and the final number of clusters with the highest score, the clustering results were mapped temporally. Since the data used for this analysis is longitudinal, some HFCAs had missing data due to the splitting and formation of new catchments over time. In these cases, the study assigned such HFCAs the archetype of their geographically nearest neighbour. Thus, for each year of the analysis, HFCAs with missing covariate data were matched to the HFCA whose polygon centroid had the shortest Euclidean distance and which had an observed archetype assignment in that year; the nearest neighbour’s archetype was then assigned to the focal HFCA. In total, 5,763 of 21,776 facility-year observations (26.5%) were assigned an archetype via this nearest-neighbour imputation. A sensitivity analysis comparing the temporal-stability and spatial-homogeneity statistics with and without imputed assignments is detailed in Additional File 7, which suggests that the findings do not change significantly due to imputation.

### Testing temporal stability and spatial homogeneity

To test whether HFCAs hold their classification more consistently under CLARA-clustering stratification than under the incidence stratification approach. We compared each HFCA’s stratum belonging from one year to the next under both methods using three measures: the retention rate (the proportion of HFCAs remaining in the same stratum), Cohen’s kappa (retention rate, adjusted for chance agreement), and the Adjusted Rand Index. For spatial homogeneity, we tested whether neighbouring HFCAs share the same stratum as their neighbour more often than would be expected by chance. We built a neighbour graph from HFCAs that share a border, and for each stratification method expressed the count of same-stratum neighbour pairs as a z-score relative to a random-stratification null. Differences between the two methods were tested with one-sided paired Wilcoxon signed-rank tests, with year-pairs (temporal) or years (spatial) as the matched units.

### Cluster feature importance and sensitivity analysis

To measure the importance of each feature, the percentage change in the silhouette score was recorded when each feature was removed from the analysis. Similarly, the individual cluster-level silhouette score percentage change was also recorded to capture the contribution of each feature to individual clusters.

## Results

### Spatio-temporal heterogeneity characterises Zambia’s malaria incidence stratification at the HFCA level, except for southern province

**Fig. 1:**
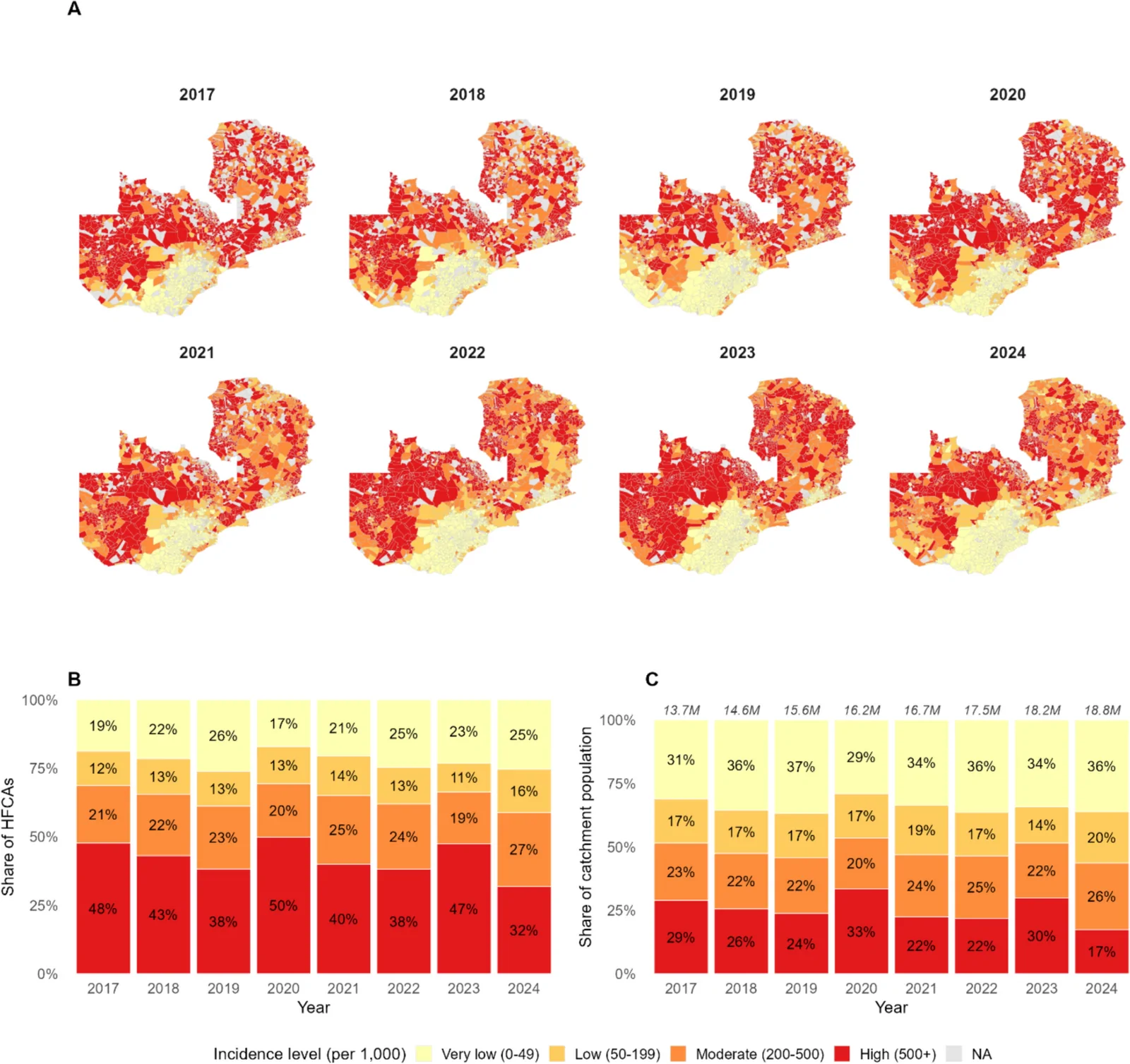
Spatio-temporal malaria incidence stratification distribution at the health facility catchment area (HFCA) level: **A** HFCAs mapped by year and categorised by malaria incidence stratification as defined by NMEC. In this figure, NA implies missing data, **B** Percentage distribution of malaria incidence stratified as defined by NMEC from 2021 to 2024. **C** Percentage of total catchment population in each incidence stratum by year

Malaria incidence stratifications, determined by the NMEC categorisation of cases per 1,000 population per year, varied heterogeneously at the HFCA level (Fig. 1). In this figure, NA implies missing data due to various reasons, such as the splitting of HFCAs. The malaria incidence trend observed in the data suggests that the southern province has historically maintained a very low incidence profile in most of its HFCAs in the analysis period (Fig. 1). Relative to the very low-risk stratum, HFCAs in other categories transition back and forth across the incidence levels over the years (Fig. 1). Additionally, as shown in Fig. 1b, malaria incidence trends have stagnated, except for 2023, when the high-risk category (500 +) increased to 47% from 38% in 2022. The stagnant trend, as shown in the figure, reflects incidence strata levels that fluctuate slightly without a consistent reduction or increase from 2017 to 2024.

### Identified and eliminated variables for cluster analysis

Table 1 presents the results from a Zero-inflated negative binomial mixed-effect regression model, with malaria incidence per 1,000 population as the dependent variable. The independent variables of interest, shown in Table 1, except for “Year”, emerged as the preliminary variables of interest after the correlation coefficient test. Thus, they were either not correlated with other variables, or they were more correlated with malaria incidence relative to the variable(s) they were correlated with, which were eliminated. The model results suggest that we failed to find a significant association between the percentage of intermittent preventive treatment in pregnancy (at least three doses, IPTp3 +), indoor residual spraying coverage, and malaria incidence at the HFCA level. Therefore, the two variables were eliminated in the subsequent cluster analysis. Nonetheless, the model identified that walking travel time to health care, percentage of malaria attributed to pregnant women, percentage of malaria attributed to children (U5), children U5 bednet coverage, bednet use rate, elevation, enhanced vegetation index, precipitation, and housing quality are highly associated with malaria incidence.

**Table 1:** Zero-inflated negative binomial mixed-effect regression model estimates for malaria incidence at the HFCA level from 2017 to 2024

Variable	IRR (95% CI)	Interpretation
Intercept	40.05 (16.54, 96.93)^***^	Intercept
Year 2018	0.882 (0.847, 0.920)^***^	vs 2017 (reference year)
Year 2019	0.858 (0.820, 0.898)^***^	vs 2017
Year 2020	1.103 (1.061, 1.147)^***^	vs 2017
Year 2021	0.865 (0.829, 0.903)^***^	vs 2017
Year 2022	0.760 (0.730, 0.792)^***^	vs 2017
Year 2023	0.958 (0.917, 1.000)	vs 2017
Year 2024	0.709 (0.672, 0.747)^***^	vs 2017
Walking travel time to health care	1.051 (1.020, 1.083)^**^	per 10% increase in minutes
Percentage of malaria attributed to pregnant women	0.458 (0.438, 0.479)^***^	per 10% increase
Percentage of malaria attributed to children (U5)	1.031 (1.010, 1.052)^**^	per 10% increase
U5 children’s bednet coverage	1.215 (1.157, 1.276)^***^	per 1-unit increase
Percent IPTp (≥ 3 doses, IPTp3 +)	1.005 (0.963, 1.049)	per 1-unit increase
Bednet use rate	1.215 (1.165, 1.266)^***^	per 10% increase
Indoor residual spraying coverage	0.999 (0.992, 1.006)	per 10% increase
Elevation	1.010 (1.008, 1.012)^***^	per 1 m increase
Enhanced vegetation index	1.107 (1.073, 1.143)^***^	per 1 SD increase
Precipitation	1.011 (1.005, 1.018)^**^	per 10% increase in mm
Percentage of good-quality housing	0.666 (0.638, 0.697)^***^	per 10% increase

Coefficients are from a zero-inflated negative binomial generalised linear mixed model with HFCA as a random intercept and Year as a fixed-effect control. Significance: ^***^p < 0.001; ^**^p < 0.01; ^*^p < 0.05. Incidence rate ratios (IRRs) are reported on each variable’s original (untransformed) scale:

Log-transformed variables (walking time, percentages of malaria in pregnant women and U5 children, bednet use rate, IRS coverage, precipitation, percentage of good-quality housing): IRR is reported per 10% increase in the original (untransformed) variable

Mean-centred variables (U5 bednet coverage, elevation): IRR is per one-unit increase in the original variable; centring affects the intercept only

Standardised variables (enhanced vegetation index): IRR is per one standard deviation increase in the original variable

Untransformed variables (IPTp3 +): IRR is per one-unit increase in the original variable

Trend-wise, the data suggest that incidence significantly differed from 2017 (the reference year) in all years except 2023. However, holding all other variables constant, the “year” variable estimates fluctuated over time, indicating the absence of a consistent trend, which statistically confirms the descriptive findings in Fig. 1.

On the other hand, both PCA and SVD methods reduced the variables from 20 to 16 at a 95% threshold (Additional File 2), compared to the nine achieved by the regression approach.

### Archetype identification and validation

Several optimal numbers of clusters, ranging from three to seven, were identified by the Hierarchical, K-means, PAMS, and CLARA methods, using data reduced by PCA, SVD, and regression. Among the four clustering methods, CLARA’s four-cluster option using regression-reduced data yielded the highest average silhouette score of 0.25, emerging as the best clustering option for defining the archetypes. In contrast, the silhouette scores for both SVD and PCA were relatively lower (0.16–0.18) than those using the nine variables, despite using the same clustering algorithms and number of clusters. As such, the regression approach was selected as the appropriate covariate reduction method.

### Identified archetypes are relatively spatially homogeneous across HFCAs compared to incidence stratification

**Fig. 2:**
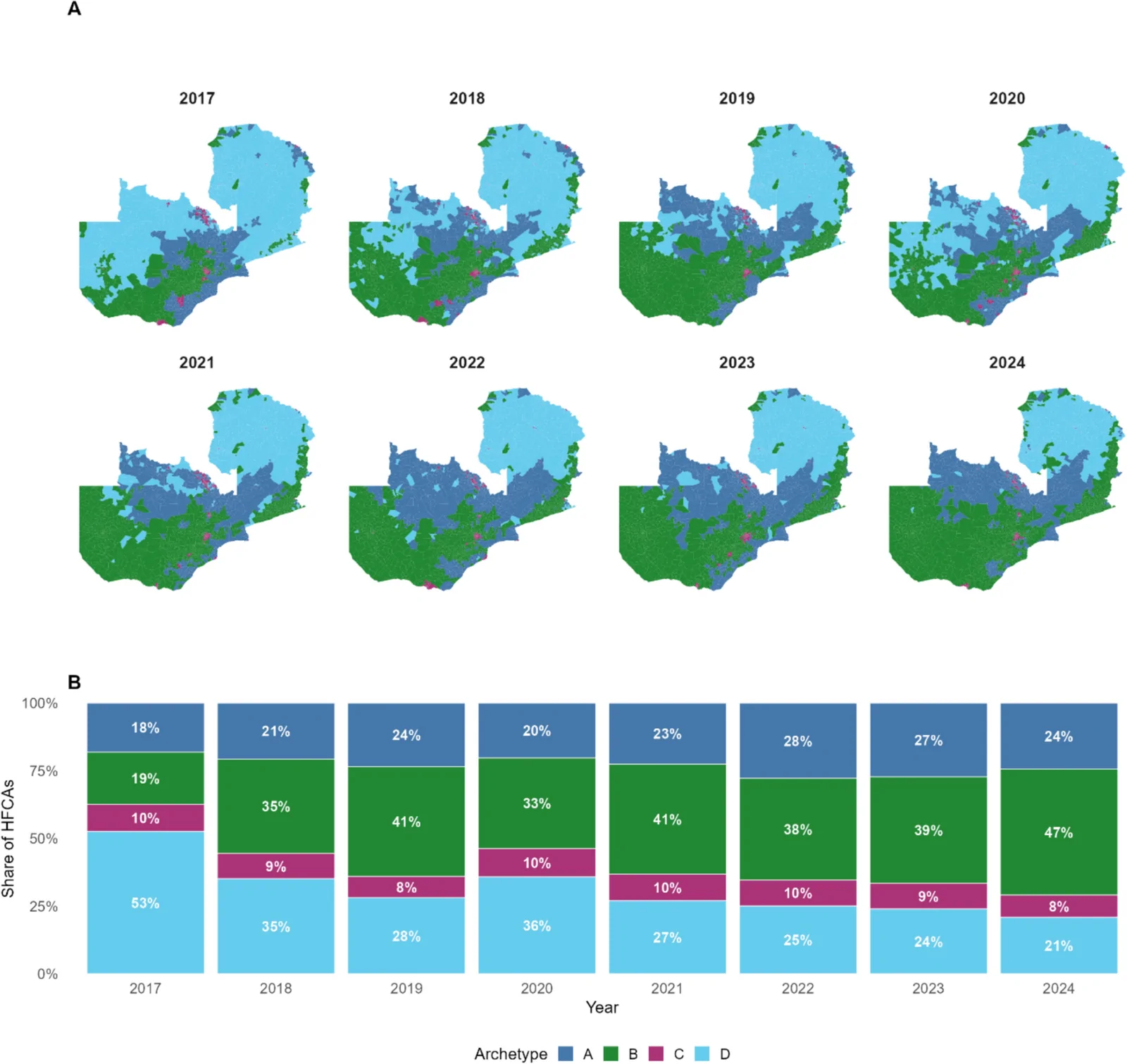
Spatio-temporal distribution of archetypes at the HFCA) level: **A** HFCAs mapped by year and categorised by archetype. **B** Archetype percentage distribution from 2021 to 2024 at HFCA level

Figure 2 shows the spatial distribution of archetypes that each HFCAs was categorised by, derived through cluster analysis from 2017 to 2024. Based on the prevailing variables associated with malaria in a particular year, some HFCAs transitioned into different archetypes. Relative to the incidence-based stratification shown in Fig. 1, the identified archetypes, as shown in Fig. 2, exhibit geographic continuity across all years, especially the most recent years. Statistically, neighbouring HFCAs shared the same archetype far more often than chance would predict, and this clustering signal was more than twice as strong as for incidence in every year (mean join-count z = 69.4 vs 32.9; *p* = 0.004; Additional File 5, Tables S1 and S3). Over the years, we observed that the majority, 38–47%, of HFCAs belonged to archetype B (Fig. 2b). We also noted that approximately one percent of the HFCAs belong to the third archetype, which visually coincides with Zambia’s major “line of rail”, a geographical landmark that defines the country’s relatively developed stretch (Fig. 2) [26].

### Identified archetypes exhibit relatively more spatio-temporal stability than incidence-based stratification

**Fig. 3:**
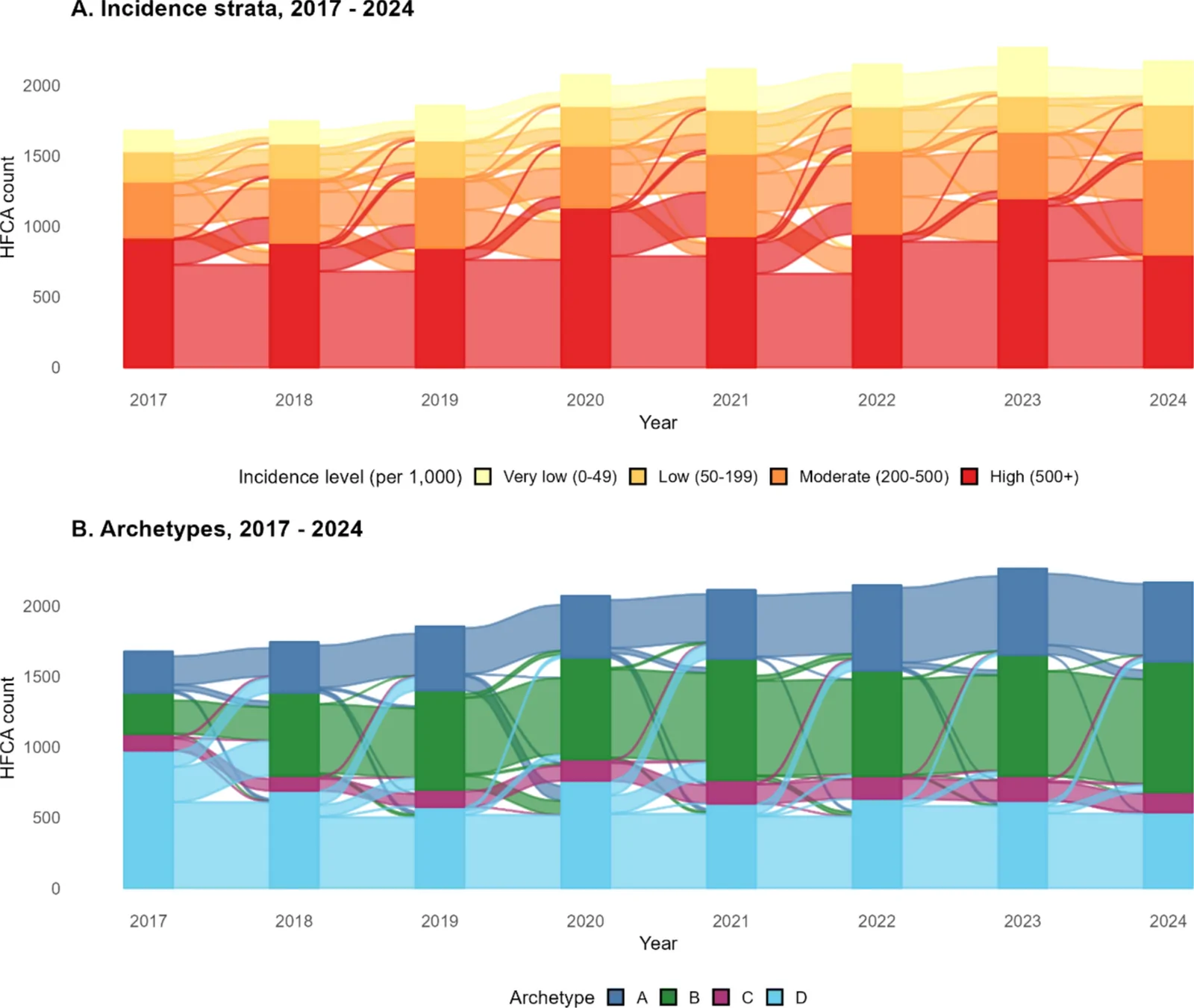
Temporal stability of health facility catchment classifications: **A** Incidence-based stratification trends from 2017 to 2024. **B** Archetype-based classification trends from 2017 to 2024

Figure 3a, b show Sankey diagrams of HFCA malaria incidence stratification and archetypes, respectively, from 2017 to 2024. In these figures, nodes (vertical bars) represent values in the respective categories for each year, and arcs represent transitions connecting nodes across years, illustrating the HFCAs’ movement from a source year to a target year. Furthermore, the size of the arcs represents the number of HFCAs that transition from the source node to the target (Fig. 3a, b). Therefore, the thicker the arc, the larger the number of HFCAs.

From 2017 to 2024, there has been an upward trend in the number of HFCAs, as some HFCAs were divided to allow the birth of new facilities. Relative to Fig. 3b, the arcs in Fig. 3a are thicker, suggesting more facility transitions between incidence levels over time than archetypes. On average, 86% of HFCAs kept the same archetype from one year to the next, compared with 67% under incidence stratification (Cohen’s κ = 0.79 vs. 0.48; ARI = 0.64 vs. 0.38; paired Wilcoxon *p* ≤ 0.016; Additional File 5, Tables S1-2). Furthermore, the probabilities for HFCAs to maintain their year-to-year transition ranged from 0.79 to 0.94 for archetypes versus 0.50 to 0.77 for incidence strata (Additional File 5, Table S1). These stability metrics suggests that archetypes are relatively more stable over time compared to incidence categorisation. Notably, we see that most of the facilities transition from the moderate (200–499) incidence level to other levels (Fig. 3a). Additionally, very few HFCAs transition into and out of the “very low (0–49) category (Fig. 3a). Similarly, almost none of the HFCAs transition from Archetype C.

### Malaria incidence stratification distribution by archetype and overall feature importance

**Fig. 4:**
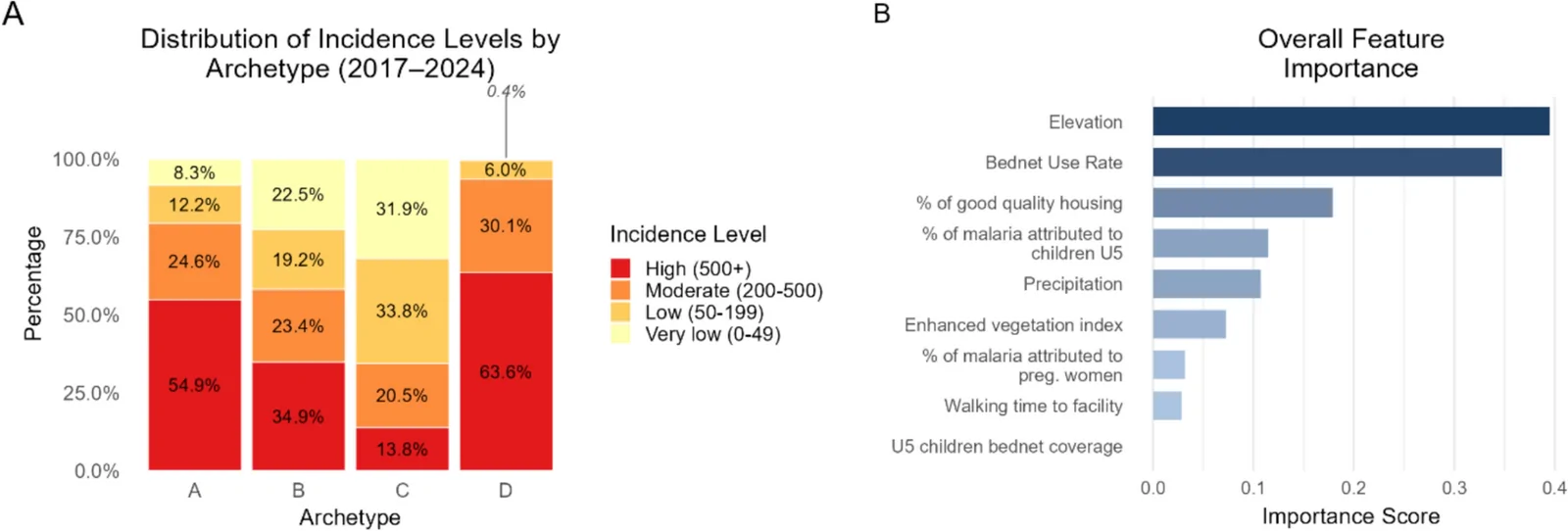
Archetype distribution among malaria incidence strata and feature importance: **A** Malaria incidence strata distribution per archetype for the analysis period 2017–2024. **B** Overall feature importance ranking in determining archetypes

Distribution-wise, all archetypes comprised HFCAs from all incidence categories (Fig. 4a). However, the proportion of very low transmission HFCAs was relatively small (only 0.4 percent) in Archetype D compared to the other archetypes (Fig. 4a). This implies that 16 distinct HFCA characteristics strata emerge if the identified archetypes are disaggregated by malaria incidence stratification (Fig. 4a). For instance, 54.9% of HFCAs belong to both Archetype A and the high-risk (500 +) malaria incidence stratum (Fig. 4a).

Figure 4b shows the overall importance ranking of features contributing to HFCA clustering. The Y-axis represents the features, while the X-axis indicates the percentage contribution each feature has on the overall clustering outcomes. As illustrated, elevation contributes approximately 39% to the clustering results (Fig. 4b). Closely following elevation is the bednet use rate at around 35%, followed by the percentage of good housing quality (Fig. 4b). Among all the features, bednet coverage for under-five children had the least contribution of approximately 2% (Fig. 4b).

### Distribution of variables associated with malaria and feature sensitivity by archetypes

**Fig. 5:**
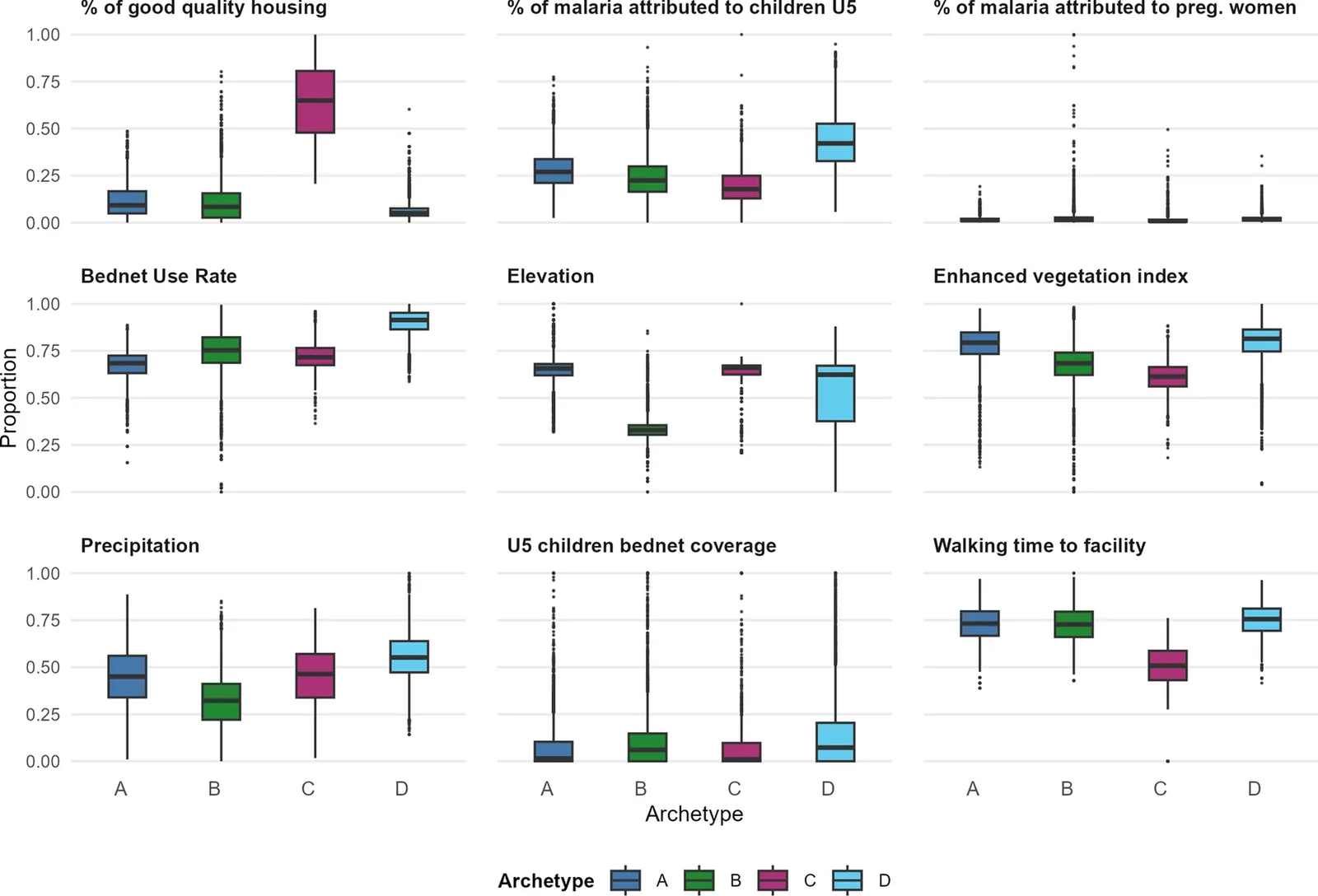
Distribution of normalised malaria-related drivers stratified by archetypes

Figure 5 summarises the distribution of variables across archetypes to characterise their composition. In Fig. 5, all variables were scaled to fall between zero and one. The contribution of each variable was also measured by its sensitivity in determining the individual archetypes, as shown in Table 2 and Additional File 4. Table 2 summarises each archetype’s profile in two related but distinct columns. Column two lists the variables along which each archetype’s distribution in Fig. 5 differs visibly from the other three in a direction consistent with promotion of malaria transmission. E.g. low housing quality, and high precipitation are listed when they distinguish the archetype from the other three; low precipitation, which would be expected to reduce transmission, is not listed where an archetype shows lower values, to avoid misattributing risk. For Archetype C, which is the protected archetype, the protective features are listed instead (high-quality housing, short walking time, low enhanced vegetation index). Column three is a subset of column two that retains only the variables with the highest absolute sensitivity scores in the per-archetype feature-importance analysis reported in Additional File 4. Column three therefore identifies, for each archetype, the variables that are both interpretable as drivers (or protective factors) of risk and contributing highly in defining the archetype’s cluster identity.

**Table 2:** Summary of archetypes by feature characteristics (value ranges) known to be promote malaria and their top-contributing features

Archetype	Malaria-relevant characterising variables (value ranges) relative to other archetypes	Top-contributing features that characterise archetype malaria transmission
A	Low housing quality Low bednet use rate High EVI Long walking time to the nearest health facility	Bednet use rate Walking time to the nearest health facility Precipitation
B	Low housing quality Low elevation Long walking time to the nearest health facility	Elevation Bednet use rate
C	High housing quality Short walking time to a health facility Low enhanced vegetation index	Housing quality Walking time to a health facility
D	Low housing quality High precipitation Low elevation Long walking time to the nearest health facility	Precipitation Elevation

Profile-wise, Fig. 5 shows that Archetypes A, B, and D share relatively low housing quality and long walking times to health facilities, in contrast to Archetype C. Within this shared pattern, Archetype A is additionally distinguished by a low bednet use rate, and its top contributing malaria-associated drivers are bednet use rate and walking time to nearest facility (Table 2 and Additional File 4). Malaria-associated variable’s characteristics in Archetype B include relatively low housing quality, low elevation, and long walking distances to the nearest health facility (Fig. 5). However, only elevation and bednet use rate are among its top-contributing features (Table 2, and Additional File 4). Archetype C is characterised by relatively low levels of all variables associated with promoting malaria (Fig. 5, Table 2, and Additional File 4). Notably, in addition to coinciding with the “line of rail”, Archetype C is also characterised by relatively high-quality housing, low EVI, and low walking time to health facility distributions. However, only housing quality and walking time to a health facility contribute highly to its clustering identity. On the other hand, Archetype D is characterised by relatively low-quality housing, high precipitation, low elevation, and long walking time to the nearest health facility (Fig. 5). Among these, precipitation and elevation are the features mostly contributing to this archetype formation (Table 2, and Additional File 4).

## Discussion

We analysed HFCA level data for Zambia. Our objectives were to identify variables associated with malaria heterogeneity at the HFCA level to cluster the HFCAs into archetypes with similar characteristics, and to compare the spatio-temporal stability between malaria incidence and archetype stratification approaches. These analyses were undertaken to build on the existing stratification approach and to inform the tailoring of malaria interventions at the HFCA level.

The analysis of malaria incidence stratification showed a non-constant trend from 2017 to 2024. Malaria spatial heterogeneity was observed nationwide at the HFCA level, except in the southern province of the country, where the majority of HFCAs had very low incidence (0–49). The very-low incidence observed in the majority of Southern province HFCAs is likely due to the deliberate scale-up of malaria control and targeted elimination efforts in the province since the 2010 s [27]. The pre-clustering variable selection process identified nine variables, which coincidentally represents access, vulnerable population, intervention history, behavioural, environmental, and socioeconomic dimensions of malaria transmission [6]. Notably, IPTp3 + and IRS coverage were not statistically associated with HFCA-level malaria incidence. We do not interpret this as evidence that these interventions are ineffective, but rather as a reflection of how each operates relative to the outcome measured. IPTp3 + targets only pregnant women, and its protective effect on maternal and neonatal outcomes is unlikely to register strongly against total HFCA-level case counts dominated by the general population. IRS, in contrast, is deliberately allocated to high-burden HFCAs in Zambia, which introduces reverse-targeting bias in an ecological cross-sectional analysis [6]. Thus, areas receiving more spray are, by design, those with higher transmission.

Relative to malaria incidence stratification, the established clusters from the identified variables exhibit geographic continuity across all years, particularly in the most recent ones, which represents a tendency of how factors such as precipitation and elevation manifest geographically [17]. Furthermore, the locations of archetypes, such as Archetype D, whose profile is characterized by high precipitation, coincided with regions (northern) known to have the highest levels of rainfall [28]. Similarly, Archetype C, which is strongly linked to good-quality housing, was identified along Zambia’s “line of rail”, a corridor of urban developed areas where such housing is concentrated [26]. The correspondence between the established archetypes and national attributes further suggest external validity of our clustering approach. Because the identified archetypes reflect recognised national attributes, they may offer a substantively useful basis for archetype-based stratification policy that is supported by three properties beyond internal cluster metrics. They are meaningful, with distinct feature profiles (Table 2; Fig. 5) that map onto recognised national attributes as described above. They are relatively stable, with HFCAs largely retaining their assignment and forming spatially contiguous regions, in contrast to the more frequent transitions observed under incidence-based stratification (Fig. 3). Additionally, they are actionable, with each archetype’s distinct feature profile pointing to a different intervention recommendation. For this, the modest silhouette score of 0.25 maybe reflecting the nature of malaria-related transmission drivers across Zambia rather than a verdict on the utility of the resulting stratification.

Except for HFCAs with very low malaria cases (0–49), a relatively higher number of HFCAs switched from one malaria incidence category to another over time, while fewer HFCAs changed their archetype. The shifts in malaria incidence classification among HFCAs may be attributed to several factors, including the adjustments to intervention strategies [6, 29, 30]. Typically, HFCAs with higher case counts receive more intensive malaria control interventions [6]. When case counts decline, these areas may transition to pre-elimination strategies [6]. However, if the new, less intensive interventions are not well matched to local conditions, or if neighbouring areas continue to have high transmission, it may lead to a resurgence in cases and a return to relatively higher-incidence strata [6, 29–31]. In contrast, the archetype-based approach offers more stability, which may inform package switching to consider the archetypical characteristics of the HFCAs, which may aid consistent progress in malaria reduction, as the interventions would be better tailored to the underlying context and needs of each HFCA [15, 17, 32]. Moreover, relying solely on malaria cases is prone to spatial error due to various reasons, such as the misclassification of patients from different catchments who prefer certain health facilities over those in their catchments [1]. The implication of such situations may result in deploying intervention packages in areas where cases are recorded, rather than in areas where they originate [1].

The distinct features characterising the established archetypes can be used to tailor interventions aimed at reducing malaria incidence. These include low bednet use and long walking time to a health facility for Archetype A; low elevation and low bednet use for Archetype B; and low elevation and high precipitation for Archetype D. For Archetype A, suggested intervention directions would include strengthening social and behaviour change communication to increase bednet use, alongside efforts to improve healthcare access where feasible, for example through the establishment of additional health posts or community-based strategies such as targeted test and treat [33, 34]. We note, however, that the observed association between longer walking time and higher malaria incidence in our cross-sectional analysis may partly reflect confounding by unmeasured ecological and socioeconomic features of rural settings that may favour transmission [35], rather than a direct causal effect of healthcare access on transmission. As, such, improvements in healthcare access alone may therefore not yield corresponding reductions in incidence without complementary interventions targeting the underlying ecological and socioeconomic drivers [7]. For Archetypes A and D, further exploration of precipitation seasonality may be necessary to ensure appropriate timing of interventions [15, 17]. In these settings, larval source management may also be considered as a supplementary intervention, specifically in areas where breeding sites are few, fixed, and findable, as recommended by the WHO. Interestingly, Archetype C has relatively few malaria-associated features contributing to it. As such, HFCAs within Archetype C could be deprioritised in terms of interventions, especially those targeting the socioeconomic determinants of malaria, since it is mainly linked with good housing quality, which may be a proxy for high socioeconomic status, while the rest of the features associated with higher malaria transmission are relatively low [19, 36, 37].

In the context of building on the current stratification approach, interventions within the control and pre-elimination packages may be prioritised based on the archetypical makeup of HFCA. This can be achieved by identifying the HFCA intersection between malaria incidence strata and archetypes, as illustrated in Additional File 3, to inform intervention allocation and prioritisation from the prescribed malaria incidence stratification packages. For example, interventions such as reactive case detection that are typically implemented in HFCAs belonging to the low and very-low malaria incidence strata can be prioritised in those that also belong to   Archetype A where access to healthcare is limited due to long walking times to health facilities [6]. Since the data used and archetypes generated are longitudinal, an averaged dataset using the same algorithm and number of clusters can be used to inform the enhancement of the malaria incidence stratification, as shown in Additional File 3. This implies that 16 distinct HFCA categories would emerge to guide the package allocation in the proposed stratification, based on cross tabulation of the four malaria incidence stratification profiles by the four newly identified archetypes.

Our approach contributes to a growing body of work using clustering methods to identify malaria transmission archetypes for intervention planning in sub-Saharan Africa. Bertozzi-Villa et al. [17], used k-means clustering on rainfall, temperature, and the relative abundance of Anopheles species to derive ten transmission archetypes across the African continent. Ozodiegwu et al. [15] used the CLARA algorithm on a similar set of climatic, entomological, and transmission-baseline variables to group Nigeria’s 774 local government areas into 22 epidemiological archetypes. Our study extends this work in three ways that reflect a different policy aim. Firstly, it is focused on a finer spatial scale (HFCA), directly tied to Zambia’s primary-care delivery network. Secondly, the variable set extends beyond climatic and entomological features to include access, behavioural, socioeconomic, and demographic dimensions, reflecting a focus on directly actionable intervention levers. Thirdly, it identifies four, which are cross-tabulated with the existing incidence-based stratification, as outlined earlier, whereas the ten and 22 archetypes by [15, 17], respectively, were used as standalone stratification outputs. Nonetheless, unsupervised clustering and climatic variables as archetype-defining features emerge common across these studies, supporting the generalisability of the archetype-based approach across sub-Saharan African settings. Differences in the number of archetypes identified and in the additional variables contributing to archetype definition reflect the prevailing feature data makeup in each setting, rather than indicating a limitation of the approach.

It is also worth noting that our study provides a precursor for more formal investigations to assess the impact of various interventions for the identified archetypes through relatively sophisticated approaches, such as mathematical models. Considering the challenges associated with historical malaria data, including issues of quality and availability at finer spatial scales [38, 39], archetypical characteristics also offer a valuable basis for projecting the outcomes of specific interventions from one HFCA to another [17]. These projections can be derived from modelling outcomes of HFCAs within the same archetype that have good-quality data available [17].

While this study provides valuable insights into archetypical makeup of HFCAs, we acknowledge certain limitations. The clustering quality, as indicated by a silhouette score of 0.25, reflects the complexity of working with high-dimensional data, which can affect clustering performance [40]. This effect was particularly evident when using relatively higher-dimensional datasets (16 features) derived from PCA and SVD, which resulted in even lower validation scores (approximately 0.15–0.18). Because referral hospitals and a subset of nested health posts lack discrete HFCA boundaries, they are not represented in our archetype assignment. Therefore, the archetypes should be interpreted as applying to HFCAs that have their own catchment area, which collectively represent 92.8% of nationally reported malaria cases over 2017–2024 (Additional File 6). Additionally, the cross-sectional nature of our analysis means that the relationships reported between covariates and malaria incidence, and between features and archetype assignment, are associational rather than causal. Despite these limitations, the current approach strikes a practical balance between clustering quality and the inclusion of a sufficient number of archetype features to meaningfully inform appropriate intervention allocation. It is also worth noting that some variables in our study were derived from modelled estimates, which may introduce a degree of uncertainty into the outcomes.

Given the multifaceted nature of malaria, this work serves as an important starting point for further refinement of archetype-based approaches. Future research could explore incorporating additional factors such as seasonality and health system characteristics, which may improve clustering outcomes. To leverage the benefits of interventions tailored to archetypes, our future work includes mathematical modelling to assess how intervention efficacy varies across different archetypes to inform the optimisation of intervention mixes. Future work could also formally examine plausible interactions between the variables associated with malaria, such as elevation with precipitation, thus, within the mathematical modelling extension where intervention and environmental effects can be assessed jointly. Additionally, further research is warranted to better understand why some HFCAs transition between malaria incidence levels over time.

## Conclusion

The study identified variables associated with malaria incidence at the HFCA level and used them to cluster HFCAs into archetypes with similar characteristics to build on the existing stratification approach. The study established that incidence-based malaria incidence stratification is unstable over time compared to archetypical characteristics. As such, the study suggests embedding the archetypical characteristics in the stratification approach to better inform intervention tailoring and prioritisation. Furthermore, the study provides a precursor for simulating potential intervention mixes based on malaria transmission archetypical characteristics, which can provide decision-makers with the anticipated results of varied investments in HFCAs with similar archetypical features.

## Supplementary Information


Additional file 1.Additional file 2.Additional file 3.Additional file 4.Additional file 5.Additional file 6.Additional file 7.

## Data Availability

Malaria incidence and intervention data are available upon request from the National Malaria Elimination Centre. Sources for public data used in the study are highlighted in the Additional File 1.
