# Supplementary material for: Archetype-based stratification to inform sub-national tailoring of malaria interventions in Zambia
Source: Malar J. 2026 Jun 18;25:312. doi: 10.1186/s12936-026-05998-7 (PMC13523243; doi:10.1186/s12936-026-05998-7)
Supplement: Supplementary file 1 [file 12936_2026_5998_MOESM1_ESM.pdf]

S1 Table: Covariate names, definitions, and sources at the health facility catchment level explored during analysis.

Variable name	Variable definition	Native spatial resolution	Source
	Dependent variable		
Number of malaria cases per 1000 population	The number of malaria cases per 1000 people aggregated per HFCA.	NA	NMEC/Zambia Health Analytics Platform [1]
	Independent variables		
	Ecological factors		
Anopheles funestus density	Estimated number of *Anopheles funestus* mosquitos in the HFCA.	~5 km × 5 km (0.0417° × 0.0417°	Malaria Atlas Project (MAP) [2,3]
Anopheles gambiae density	Estimated number of *Anopheles gambiae* mosquitos in the HFCA.	~5 km × 5 km (0.0417° × 0.0417°	MAP[2,3]
Enhanced vegetation index	Enhanced vegetation index for quantifying vegetation greenness in units of the spectral index	~5 km × 5 km (0.0417° × 0.0417°	MAP [4,5]
	Socioeconomic and demographic		
% good quality housing	Percentage of housing units in the HFCA is classified as improved.	~5 km × 5 km (0.0417° × 0.0417°	MAP [3,6]
% with higher education	Percentage of individuals in the HFCA with at least higher education.	NA	DHS [7] and MIS 2021 [communication with NMEC]
Population density	Predicted population density in an HFCA	~1 km × 1 km (0.0083° × 0.0083°)	Center for International Earth Science Information Network, Columbia University or Humanitarian Data Exchange [8]
Average household size	Predicted average number of household members in the HFCA.	NA	DHS [7]and MIS 2021 [communication with NMEC]
	Environmental		
Elevation	HFCA elevation above sea level in meters	~10 km × 10 km (0.083° × 0.083°)	Multi-Error-Removed Improved-Terrain DEM [9]

Total precipitation	Estimated total amount of water measured in meters if spread evenly over HFCA measured in meters.	~5.5 km × 5.5 km (0.05° × 0.05°)	Climate Hazards Center InfraRed Precipitation with Station data (CHIRPS v3.0) [10,11]
Temperature	Estimated temperature in the air in the HFCA in degrees Celsius.	~28 km × 28 km (0.25° × 0.25°)	European Center for Medium Range Weather Forecasts (ECMWF), Climate Data Store [12]
Relative Humidity	Relative Humidity	~28 km × 28 km (0.25° × 0.25°)	European Center for Medium Range Weather Forecasts (ECMWF), Climate Data Store [12]
	Behavioural		
Bed net use	Estimated percentage of individuals in the HFCA that use bed net.	~5 km × 5 km (0.0417° × 0.0417°)	MAP [3,13]
Health-seeking behaviour	Estimated percentage of children whose care providers sought medical attention from a professional health care provider after experiencing a fever.	NA	DHS [7]and MIS 2021 [communication with NMEC]
Antenatal care (ANC) coverage	Estimated percentage of women aged 15-49 with a live birth in a year that received antenatal care four or more times who live in the HFCA.	NA	NMEC/Zambia Health Analytics Platform [1]
	Health system		
Bed net coverage	Predicted percentage of individuals with access to bed nets.	NA	NMEC/Zambia Health Analytics Platform [1]
Yearly IRS coverage	Estimated or predicted yearly IRS coverage in the HFCA.	NA	NMEC/Zambia Health Analytics Platform [1] and MAP [3]
Number of CHAs	Humber of community health assistants in the HFCA that were active in a year.	NA	NMEC/Zambia Health Analytics Platform [1]

IPTp	Intermittent preventive treatment in pregnancy	NA	NMEC/Zambia Health Analytics Platform [1]
	Access		
Distance to nearest neighbouring facility	Distance to nearest neighbouring facility calculated using facility coordinates	NA	NMEC/Zambia Health Analytics Platform [1]
Motorised travel time to health care in minutes	Predicted travel time by a motorised form of transport to a healthcare facility in minutes	~5 km × 5 km (0.0417° × 0.0417°)	MAP [3]
Walking travel time to health care in minutes	Predicted walking travel time to health care in minutes	~5 km × 5 km (0.0417° × 0.0417°)	MAP [3]

Variable transformation and regression procedure.

Continuous predictors were transformed prior to model fitting to address skewness and to place variables on comparable scales for variable selection. Walking time to the nearest health facility, the percentages of malaria attributed to pregnant women and to children under five, bednet use rate, IRS coverage, precipitation, and the percentage of good-quality housing were log-transformed; U5 bednet coverage and elevation were mean-centred; the enhanced vegetation index was standardised (z-scored); and the percentage of pregnant women receiving at least three doses of IPTp was retained on its native scale.

The outcome variable was the integer-rounded incidence per 1000 population per HFCA per year, modelled with a negative binomial likelihood. Several candidate specifications were fitted and compared, including standard and zero-inflated negative binomial models and a Poisson alternative, with random intercepts at the HFCA and at the province level. The final model was selected on the basis of the lowest Akaike information criterion (AIC) and comprised a zero-inflated negative binomial generalised linear mixed model with HFCA as a random intercept and Year as a fixed-effect control. Coefficients are reported as incidence rate ratios (IRRs) on each predictor's original (untransformed) scale, computed as $\exp(\beta \times \log(1.10))$ for log-transformed predictors (i.e. per 10% increase in the original variable), $\exp(\beta)$ for mean-centred and untransformed predictors (per one-unit increase in the original variable), and $\exp(\beta)$ for standardised predictors (per one standard deviation increase in the original variable), with corresponding 95% confidence intervals.

References

1. Ministry of Health. https://zhap.zenysis.com/overview.

2. Sinka ME, Bangs MJ, Manguin S, Rubio-Palis Y, Chareonviriyaphap T, Coetzee M, et al. A global map of dominant malaria vectors. Parasit Vectors. 2012;5: 1–11. doi:10.1186/1756-3305-5-69/FIGURES/7

3. Malaria Atlas Project | Home. [cited 22 May 2025]. Available: https://malariaatlas.org/

4. Oxford MAP EVI: Malaria Atlas Project Gap-Filled Enhanced Vegetation Index | Earth Engine Data Catalog | Google for Developers. [cited 2 Jun 2025]. Available: https://developers.google.com/earth-engine/datasets/catalog/Oxford_MAP_EVI_5km_Monthly

5. Weiss DJ, Atkinson PM, Bhatt S, Mappin B, Hay SI, Gething PW. An effective approach for gap-filling continental scale remotely sensed time-series. ISPRS Journal of Photogrammetry and Remote Sensing. 2014;98: 106–118. doi:10.1016/J.ISPRSJPRS.2014.10.001

6. Tusting LS, Bisanzio D, Alabaster G, Cameron E, Cibulskis R, Davies M, et al. Mapping changes in housing in sub-Saharan Africa from 2000 to 2015. Nature 2019 568:7752. 2019;568: 391–394. doi:10.1038/s41586-019-1050-5

7. A ZSA-ZS, H M of H-MO, UTH-VL UTHVL-, F IC. Zambia Demographic and Health Survey 2018. Lusaka, Zambia: ZSA, MOH, UTH-VL and ICF; 2020. Available: https://www.dhsprogram.com/pubs/pdf/FR361/FR361.pdf

8. Humanitarian Data Exchange | Find & Use Crisis Data | HDX. [cited 22 May 2025]. Available: https://data.humdata.org/

9. Yamazaki D, Ikeshima D, Tawatari R, Yamaguchi T, O'Loughlin F, Neal JC, et al. A high-accuracy map of global terrain elevations. Geophys Res Lett. 2017;44: 5844–5853. doi:10.1002/2017GL072874;WGROUP:STRING:PUBLICATION

10. Data Sets | Climate Hazards Center - UC Santa Barbara. [cited 22 May 2025]. Available: https://www.chc.ucsb.edu/data

11. Funk C, Peterson P, Landsfeld M, Pedreros D, Verdin J, Shukla S, et al. The climate hazards infrared precipitation with stations—a new environmental record for monitoring extremes. Scientific Data 2015 2:1. 2015;2: 1–21. doi:10.1038/sdata.2015.66

12. J Muñoz Sabater. ERA5-Land monthly averaged data from 1950 to present. Copernicus Climate Change Service (C3S) Climate Data Store (CDS). (Accessed on 25-12-2024). 2019.

13. Bertozzi-Villa A, Bever CA, Koenker H, Weiss DJ, Vargas-Ruiz C, Nandi AK, et al. Maps and metrics of insecticide-treated net access, use, and nets-per-capita in Africa from 2000-2020. Nature Communications 2021 12:1. 2021;12: 1–12. doi:10.1038/s41467-021-23707-7
