# Supplementary material for: Archetype-based stratification to inform sub-national tailoring of malaria interventions in Zambia
Source: Malar J. 2026 Jun 18;25:312. doi: 10.1186/s12936-026-05998-7 (PMC13523243; doi:10.1186/s12936-026-05998-7)
Supplement: Supplementary file 2 [file 12936_2026_5998_MOESM3_ESM.pdf]

**Additional File 3: Enhanced Malaria Risk Stratification with Archetypical Characteristics**

Additional File 3 - Figure 1 presents faceted intersections of malaria risk strata and archetypes to illustrate how archetypical characteristics may enhance the incidence-based stratification. The figure is based on 2024 malaria risk stratification and four archetypes identified using the average data from 20217 to 2024. For example, 'High (500+) – 1' in the figure refers to HFCAs that fall within the high malaria risk stratum and belong to Archetype A (Additional File 3 - Figure 1). As such, 16 unique profiles result from using both malaria risk and archetype stratification approaches (Additional File 3 - Figure 1). From the figure, we note that the HFCAs in the high malaria risk stratum (row 1) are influenced by various sets of factors that differ from one another depending on the archetype to which they belong, despite all belonging to the same malaria risk category Additional File 3 - Figure 1. A similar trend is observed in the moderate and low risk stratum. Additionally, the majority of the HFCAs in the very-low malaria risk stratum (row 4) belong to Archetype B, which is influenced by low elevation and bednet use rates (Additional File 3 - Figure 1 and Table 2).

The output map showing the archetypes only, generated from averaged data, is presented in Additional File 3 - Figure 2.

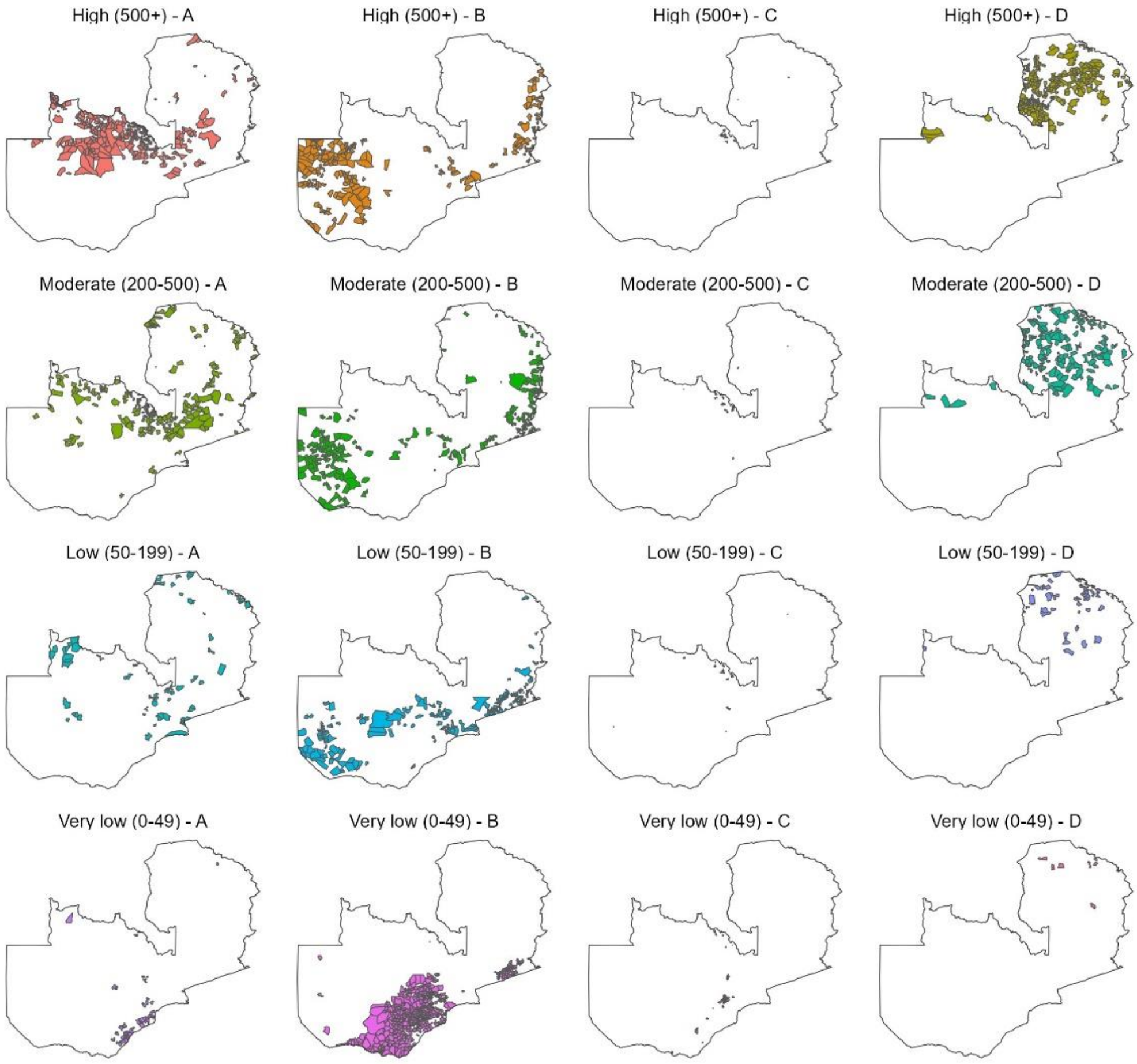


*Additional File 3 - Figure 1: Faceted overlay of malaria risk stratification and archetypes: The plots used 2024 malaria risk stratification and archetypes from the averaged data from 2017-2024.*

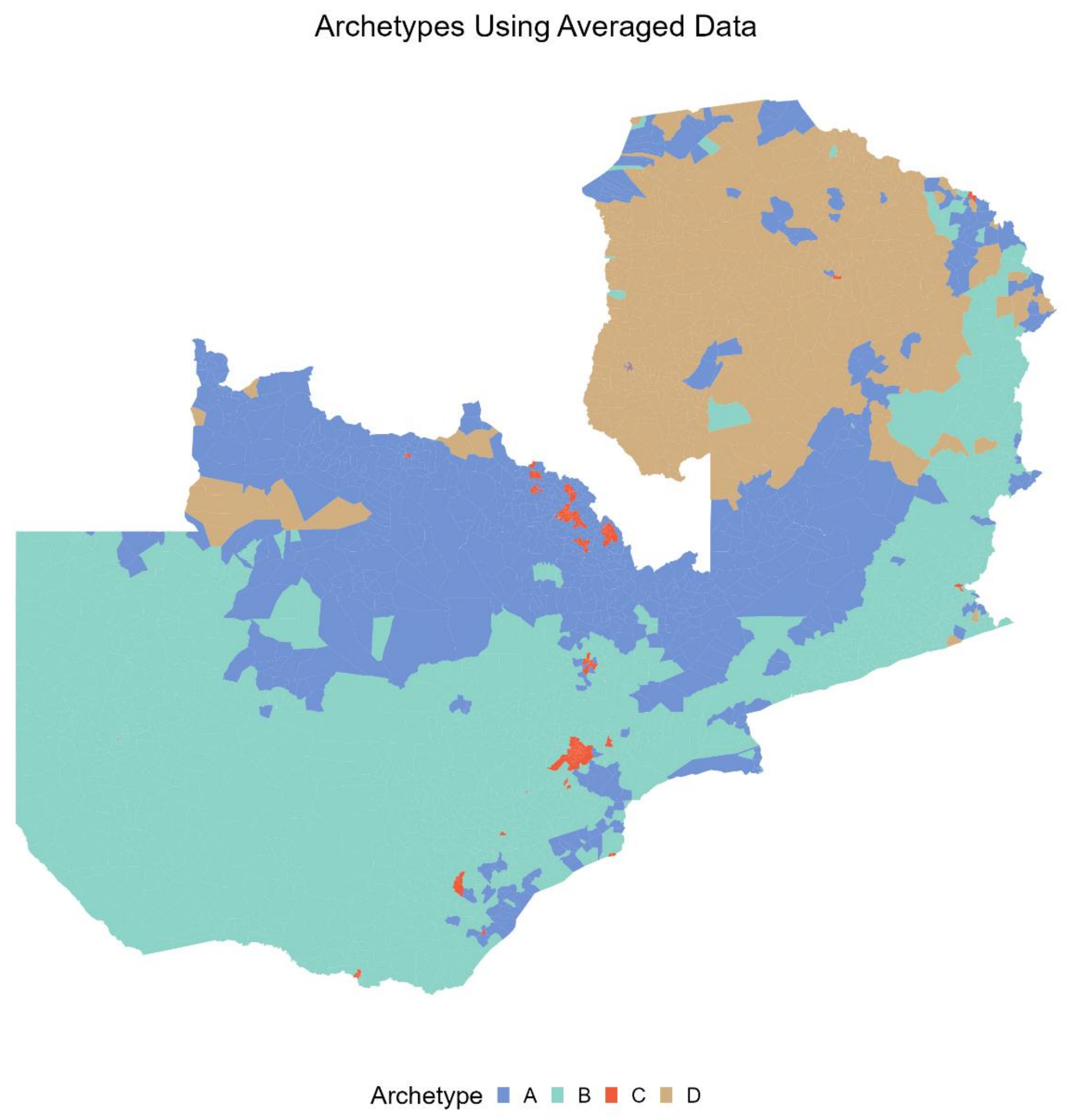


*Additional File 3 - Fig 2: Archetypes identified by cluster analysis using the averaged data from 2017 to 2024 using the CLARA algorithm.*
