# Supplementary material for: Archetype-based stratification to inform sub-national tailoring of malaria interventions in Zambia
Source: Malar J. 2026 Jun 18;25:312. doi: 10.1186/s12936-026-05998-7 (PMC13523243; doi:10.1186/s12936-026-05998-7)
Supplement: Supplementary file 3 [file 12936_2026_5998_MOESM5_ESM.pdf]

Additional File 5: Tests of Archetype Stability and Spatial Homogeneity

This supplementary material presents temporally stability and spatially homogeneous of the identified archetypes in comparison to the incidence-based stratification. Three tests are reported. Temporal stability is measured per stratification method as the agreement between HFCA strata belonging in consecutive years, summarised by the retention rate, Cohen's kappa, and the Adjusted Rand Index (ARI). Spatial homogeneity is measured per stratification method per year using the global join-count statistic against a random-stratification null, reported as a z-score on a queen-contiguity neighbour graph computed once on the full HFCA shapefile; both stratification methods were evaluated on the same subset of HFCAs each year (those with non-missing strata for both stratification methods) using identical spatial weights. Differences between stratification methods were tested using one-sided paired Wilcoxon signed-rank tests, with year-pairs (temporal) or years (spatial) as the matched units.

Table S1. Summary of formal stability tests

Mean values across all year-pairs (temporal metrics) or all years (spatial z-score), with one-sided paired Wilcoxon p-values testing the hypothesis that the archetype stratification method exceeds the incidence stratification method.

Metric	Archetype	Incidence	Wilcoxon p (one-sided)
Mean year-on-year retention	0.856	0.665	0.008
Mean year-on-year Cohen's kappa	0.791	0.481	0.008
Mean year-on-year Adjusted Rand Index	0.637	0.384	0.016
Mean yearly spatial z-score (join count)	69.40	32.86	0.004

Table S2. Year-on-year temporal stability metrics, by stratification method

Retention, Cohen's kappa, and Adjusted Rand Index for each consecutive year pair, computed on HFCAs with non-missing strata belonging in both years. Higher values indicate more stable classification.

Note. Wilcoxon p-values at or near 0.0078 and 0.0039 indicate that the archetype stratification method outperformed the incidence stratification method in every paired comparison; the p-value of 0.016 for the ARI metric reflects one year-pair (2017–2018) in which the ranking went the other way.

Stratification method	Year pair	n HFCAs	Retention	Cohen's κ	ARI
Archetype	2017-2018	1561	0.725	0.584	0.354
Incidence	2017-2018	1561	0.703	0.507	0.424
Archetype	2018-2019	1628	0.848	0.781	0.607

Stratification method	Year pair	n HFCAs	Retention	Cohen's κ	ARI
Incidence	2018-2019	1628	0.674	0.484	0.369
Archetype	2019-2020	1802	0.829	0.755	0.567
Incidence	2019-2020	1802	0.629	0.412	0.355
Archetype	2020-2021	1955	0.830	0.757	0.572
Incidence	2020-2021	1955	0.651	0.462	0.374
Archetype	2021-2022	1933	0.907	0.869	0.762
Incidence	2021-2022	1933	0.625	0.443	0.311
Archetype	2022-2023	2066	0.937	0.911	0.826
Incidence	2022-2023	2066	0.730	0.585	0.488
Archetype	2023-2024	2084	0.916	0.879	0.772
Incidence	2023-2024	2084	0.643	0.475	0.366

Table S3. Yearly spatial homogeneity (join-count z-scores)

Global same-category join-count z-scores per stratification method per year, against a random-stratification belonging null on a queen-contiguity neighbour graph. Higher z indicates stronger spatial clustering of like-strata HFCAs. Both stratification methods were evaluated on the same subset of HFCAs each year.

Year	n HFCAs	Archetype z	Incidence z
2017	1674	44.87	23.11
2018	1741	53.82	24.46
2019	1851	65.35	25.38
2020	2068	65.84	37.10
2021	2110	75.45	34.76
2022	2143	83.81	39.46
2023	2262	85.94	42.81
2024	2164	80.13	35.77

Year-to-year Transition Probability Matrices.

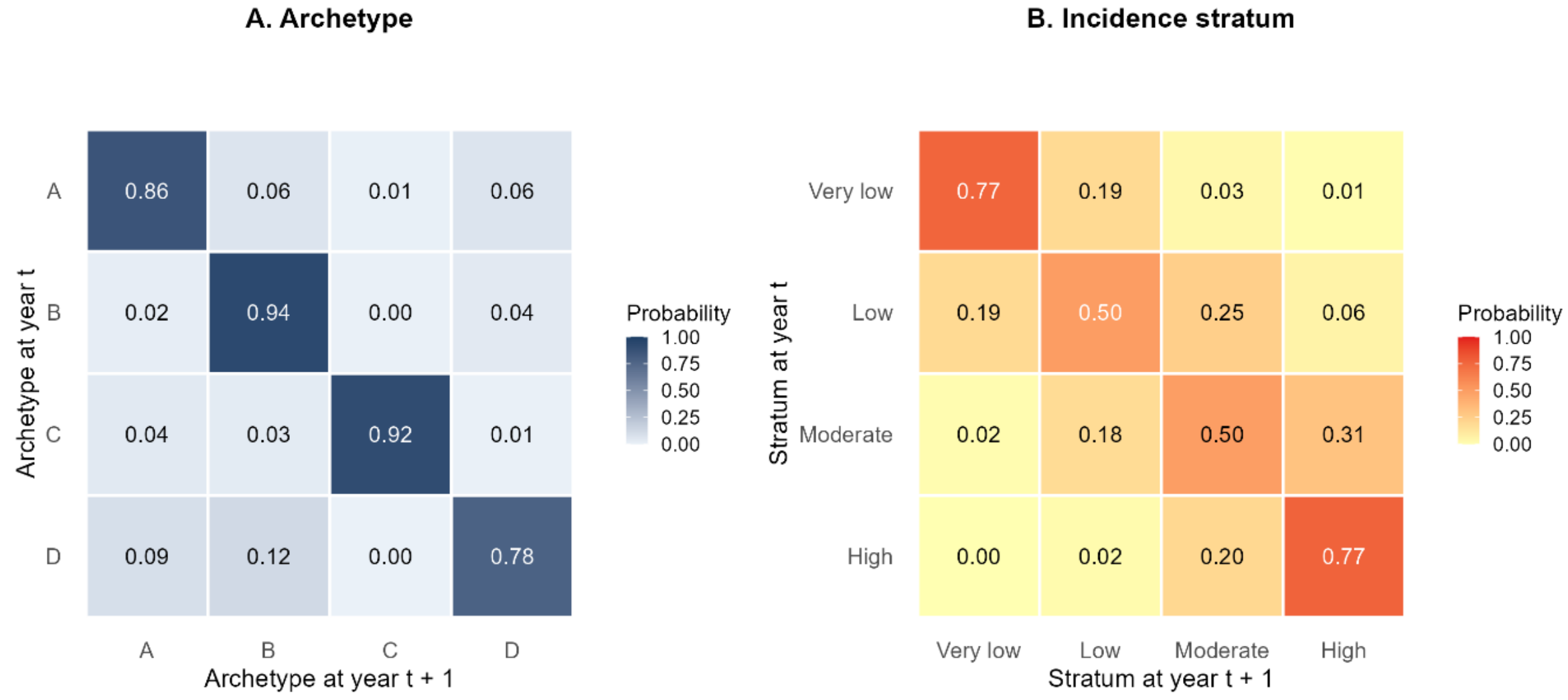

Figure S1: Overall year-to-year transition probability matrices for the archetype (A) and incidence (B) classifications. Each cell is the overall probability that an HFCA in the row category at year t was in the column category at year t + 1.

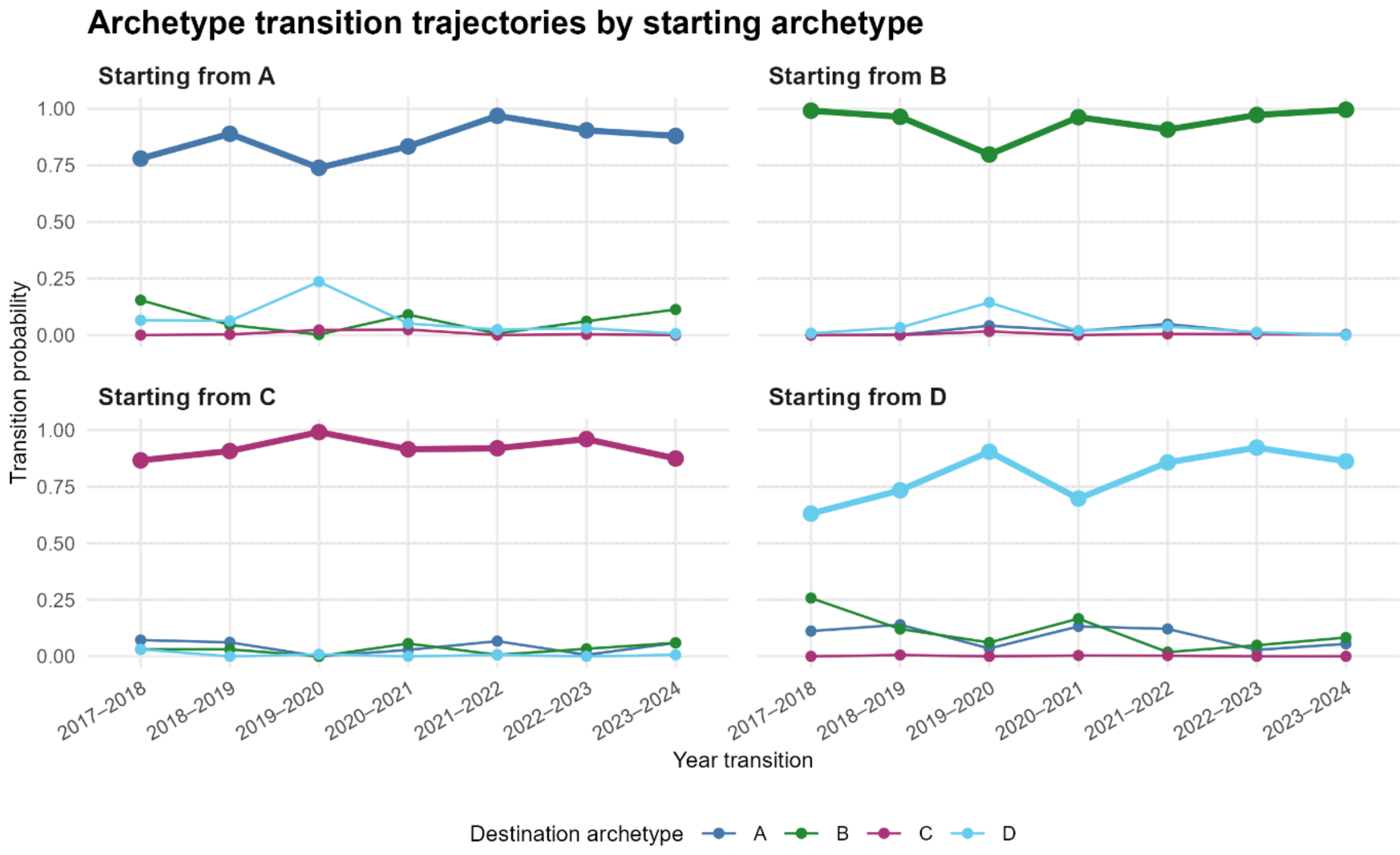

Figure S2: Year-to-year transition trajectories for the archetype classification. Each panel shows the probability that an HFCA in the starting archetype (panel title) was classified in each destination archetype one year later, across the seven consecutive year-pair transitions. The thicker line in each panel is the self-retention trajectory; the three thinner lines show switch probabilities to the other archetypes. Probabilities within each panel and year-pair sum to one.

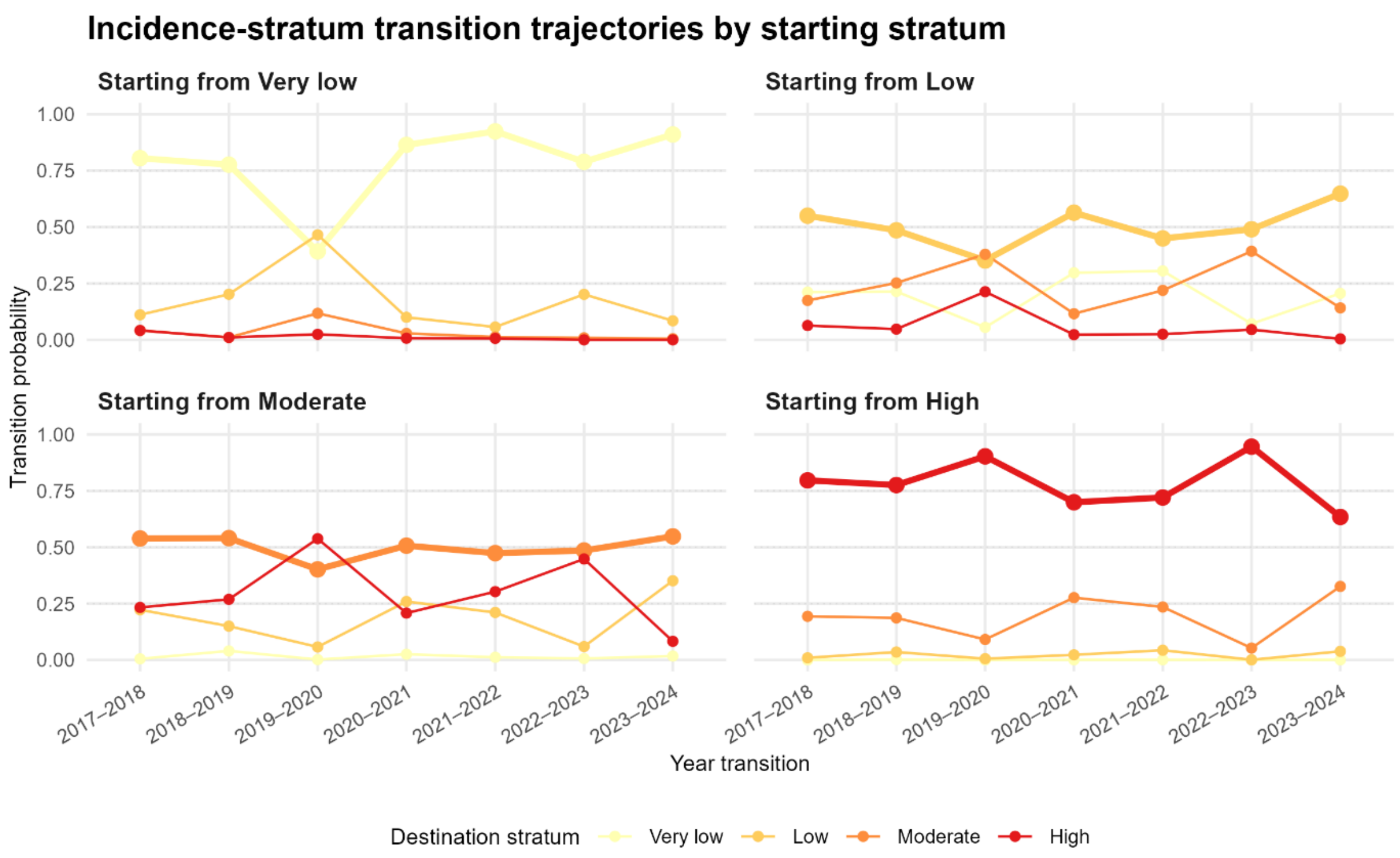

Figure S3: Year-to-year transition trajectories for the archetype classification. Each panel shows the probability that an HFCA in the starting incidence level (panel title) was classified in each destination incidence level one year later, across the seven consecutive year-pair transitions. The thicker line in each panel is the self-retention trajectory; the three thinner lines show switch probabilities to the other archetypes. Probabilities within each panel and year-pair sum to one.
