# Supplementary material for: Archetype-based stratification to inform sub-national tailoring of malaria interventions in Zambia
Source: Malar J. 2026 Jun 18;25:312. doi: 10.1186/s12936-026-05998-7 (PMC13523243; doi:10.1186/s12936-026-05998-7)
Supplement: Supplementary file 4 [file 12936_2026_5998_MOESM6_ESM.pdf]

Additional File 6

Characterizing HFCAs included versus excluded for analysis

Background

This Additional File characterises the excluded HFCAs against those retained for analysis to assess whether the exclusions are geographically or epidemiologically systematic in ways that could affect the interpretation of the identified archetypes.

Three-tier accounting of HFCAs

Of the 3,285 unique HFCAs identified in HMIS during 2017- 2024, 382 did not report (i.e their record was NA) malaria incidence over the analysis period and are therefore not part of the analysis dataset regardless of geography. Among the 2,903 HFCAs that reported incidence, 2,568 (88.5%) had a matching HFCA shapefile boundary and were retained for analysis, and 335 (11.5%) lacked a matching boundary and were excluded. A further 154 shapefile boundaries had no HMIS reporting facility during the period and therefore weren't included.

Table S1. Three-tier accounting of HFCAs in the analysis (2017-2024).

Tier	Group	N
1. HMIS dataset	All unique HFCAs in HMIS, 2017-2024	3,285
2. Data filter	HFCAs without malaria incidence (excluded a priori)	382
2. Data filter	HFCAs with malaria incidence (analysis dataset)	2,903
3. Geographic match	HFCAs with matching HFCA shapefile boundary (RETAINED)	2,568
3. Geographic match	HFCAs without matching shapefile boundary (EXCLUDED)	335
4. Shapefile side	HFCA shapefile boundaries in total	2,722
4. Shapefile side	HFCAs in Shapefile boundaries without HMIS reporting facility in 2017- 2024	154

Facility-type composition

Rural Health Posts: balanced between included (1,164; 45.3% of the retained set) and excluded (152; 45.4% of the excluded set), with an exclusion rate of 11.6%.

Rural Health Centres: exclusion rate of only 4.1%.

Hospital-type facilities: are heavily concentrated in the excluded set, consistent with the third reason for exclusion stated in the Methods: 90.9% (1/11) of District/Referral Hospitals. Of the 11 hospital only Luangwa District Hospital has catchment area. Additionally, 83.3% of Mini

Hospitals, 70.6% of "Other" hospitals, and 42.9% of Mission Hospitals lack a formal catchment boundary because majority of them function as referral facilities.

Urban Health Posts: show a moderate over-representation in the excluded set (32.1% exclusion rate), reflecting the categorization of health posts not having functioning as subset of health centers, therefore a number of them not having own catchment boundaries.

Overall: The association between facility type and exclusion status is large (Cramér's V = 0.38, $p < 0.001$), but this is exactly what the stated exclusion criterion predicts. Referral hospitals were excluded because they do not have catchment areas, so a test of whether facility type predicts exclusion is, in effect, a test of whether the exclusion rule was applied consistently. However, when we examine whether the included and excluded facilities differ in reported malaria burden, the two groups are essentially indistinguishable (standardised mean difference = −0.08; near-identical NMEC stratum distributions)

Table S2. Facility-type composition of included and excluded HFCAs after MFL-based Urban/Rural disambiguation.

Facility type	Excluded (n)	Included (n)	Total	% excluded
Rural Health Post	152	1,164	1,316	11.6%
Rural Health Centre	45	1,051	1,096	4.1%
Urban Health Centre	31	204	235	13.2%
Urban Health Post	42	89	131	32.1%
Hospital-affiliated Health Centre	10	32	42	23.8%
Mini Hospital	25	5	30	83.3%
Other Hospital	12	5	17	70.6%
Health Post (unspecified location)	5	10	15	33.3%
District/Referral Hospital	10	1	11	90.9%
Mission Hospital	3	4	7	42.9%
Unclassified	0	2	2	0.0%
Health Centre (unspecified location)	0	1	1	0.0%
Total	335	2,568	2,903	11.5%

Geographic distribution

Exclusion rates vary across provinces but are not strongly skewed ($\chi^2 = 41.0$, df = 9, $p = 5.1 \times 10^{-6}$; Cramér's V = 0.12). The two most urbanised provinces, Lusaka (21.8%) and Copperbelt (14.1%) show the highest exclusion rates, consistent with the concentration of referral hospitals along Zambia's "line of rail". The lowest exclusion rates are in Western (7.3%) and Eastern (8.2%) provinces, both predominantly served by Rural Health Centres and Rural Health Posts that retain their own catchment boundaries.

Table S3. Geographic (province) distribution of included and excluded HFCAs.

Province	Excluded (n)	Included (n)	Total	% excluded
Lusaka	43	154	197	21.8%
Luapula	44	236	280	15.7%
Copperbelt	43	263	306	14.1%
Muchinga	19	127	146	13.0%
North Western	37	268	305	12.1%
Northern	31	265	296	10.5%
Central	33	288	321	10.3%
Southern	33	351	384	8.6%
Eastern	30	337	367	8.2%
Western	22	279	301	7.3%
Total	335	2,568	2,903	11.5%

Reported malaria burden

The distribution of reported HMIS malaria incidence per 1,000 population during 2017-2024 was very similar between included and excluded HFCAs (mean 693 vs 641; median 627 vs 464). The standardised mean difference (SMD) of -0.08 is well below the conventional $|SMD| < 0.10$ threshold for negligible imbalance, and the kernel density estimates overlap substantially over the full range of incidence (Figure S1C). The Wilcoxon rank-sum test reaches conventional statistical significance ($p = 1.1 \times 10^{-3}$), but this may reflect the large sample size (n = 2,903) rather than a meaningful effect.

Stratifying HFCAs by the four NMEC incidence categories shows that both groups contain HFCAs from all strata (Figure S1D). The excluded set has a modest skew toward the "very low" stratum (25.4% vs 16.7% in the included set) and is slightly under-represented in the "high" stratum (47.5% vs 58.2%), consistent with the over-representation of urban referral hospitals in the excluded set since transmission is lower in urban areas. The association is

statistically significant (p = 0.00022) but the effect size is small (Cramér's V = 0.08). With n = 2,903, even tiny differences become significant, so the p-value only tells us the distributions are not identical.

Table S4. Distribution of HFCAs across NMEC malaria incidence strata.

NMEC stratum	Excluded (n)	Excluded (%)	Included (n)	Included (%)
Very low (0–49)	85	25.4%	428	16.7%
Low (50–199)	38	11.3%	254	9.9%
Moderate (200–499)	53	15.8%	391	15.2%
High (500+)	159	47.5%	1,495	58.2%
Total	335	100%	2,568	100%

National burden coverage

Despite accounting for 11.5% of facilities in the analysis dataset, the 335 excluded HFCAs contributed only 7.2% of total HMIS-reported malaria cases over 2017–2024. The 2,568 retained HFCAs captured 92.8% of the national reported burden, indicating that the archetypes describe the geographic distribution of the majprity of Zambia's malaria burden.

Table S5. National burden coverage by inclusion status, 2017-2024.

Status	Total cases reported (2017-2024)	% of national total
Included (retained for analysis)	43,539,357	92.8%
Excluded (no shapefile boundary)	3,391,120	7.2%
Total (analysis dataset)	46,930,477	100%

Formal statistical tests

Table S6 summarises the tests and effect sizes used to assess differences between included and excluded HFCAs. Effect sizes (Cramér's V, standardised mean difference) are reported alongside p-values because the large sample size makes even trivial differences statistically significant; the effect sizes are the substantive signal.

Table S6. Formal statistical tests comparing included and excluded HFCAs.

Variable compared	Test	Statistic	df	p-value	Effect size
Province	χ^2 test	41.0	9	5.1×10^{-6}	Cramér's V = 0.12
Facility type	χ^2 test	412	11	< 0.001	Cramér's V = 0.38
NMEC incidence stratum	χ^2 test	19.4	3	2.2×10^{-4}	Cramér's V = 0.08
Mean incidence per 1,000	Wilcoxon rank-sum	382,968	-	1.1×10^{-3}	SMD = −0.08

Summary figure

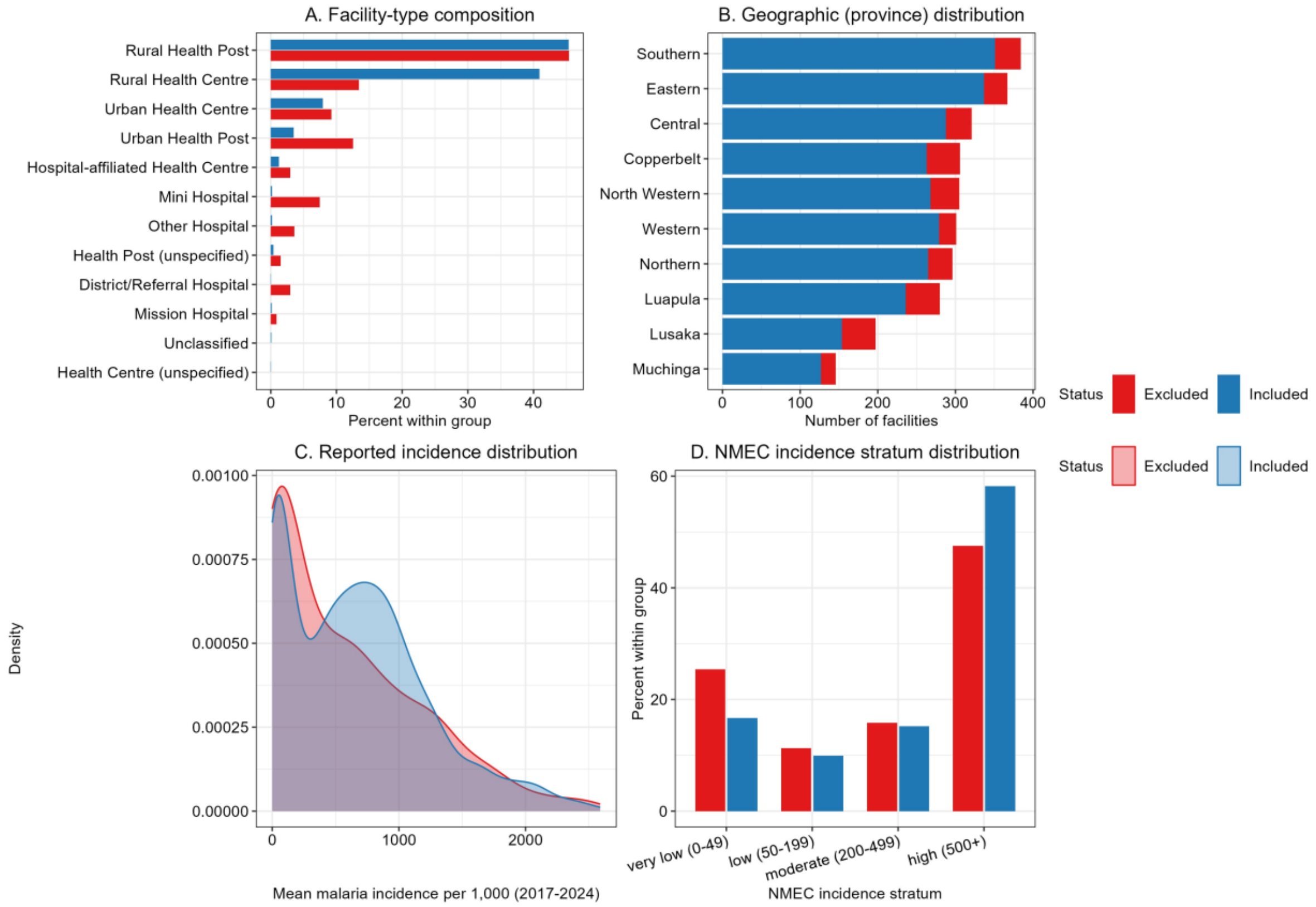

Figure S1. Four-panel comparison of included and excluded HFCAs. (A) Facility-type composition (percentage within each group); Rural Health Posts are essentially balanced between groups (≈45% in both), hospital-type facilities are heavily over-represented in the excluded set, and Urban Health Posts are moderately over-represented in the excluded set. (B) Geographic (province) distribution, showing higher absolute counts of exclusions in Lusaka, Luapula, and Copperbelt provinces. (C) Kernel density of mean HMIS malaria incidence per 1,000 population (2017-2024); the two distributions overlap substantially across the full range. (D) Distribution across the four NMEC incidence strata; both groups contain HFCAs from all strata, with a modest skew of excluded HFCAs toward the "very low" stratum.

Conclusion

First, Rural Health Posts are essentially balanced between the two groups, suggesting that the archetypes generalise well to community-level rural facilities. Second, the excluded set is over-represented in hospital-type facilities (Cramér's V = 0.38), but this is by construction: referral hospitals in the Zambian primary-care system do not have catchment areas and would not be meaningful units for any archetype analysis. Third, despite these compositional differences the two groups are essentially indistinguishable in terms of reported malaria burden (SMD = -0.08; nearly identical incidence-stratum distributions) and the retained set captures 92.8% of the national reported case burden. We therefore conclude that the four archetypes maybe considered representative of the HFCAs that have their own catchment area and account for the majority of Zambia's malaria burden. Also, the archetypes should not be interpreted as describing referral hospitals, which by design do not have catchment areas in the Zambian primary-care system.
