# Supplementary material for: Archetype-based stratification to inform sub-national tailoring of malaria interventions in Zambia
Source: Malar J. 2026 Jun 18;25:312. doi: 10.1186/s12936-026-05998-7 (PMC13523243; doi:10.1186/s12936-026-05998-7)
Supplement: Supplementary file 5 [file 12936_2026_5998_MOESM7_ESM.pdf]

Additional File 7

Sensitivity analysis: impact of nearest-neighbour imputation on archetype assignments

Background

In the main analysis, HFCAs whose covariate data were unavailable in a given year were assigned the archetype of their nearest neighbour (Euclidean distance between centroids), as described in the Methods. This Additional File quantifies the extent of the imputation, characterises whether it is geographically or temporally systematic, and presents a sensitivity analysis to assess whether the imputation procedure biases the temporal stability and spatial clustering of the identified archetypes. The imputation was applied to archetype assignments only; HMIS-reported malaria incidence values are not imputed and are used as observed.

Proportion of HFCAs affected

Across the 2,722 unique HFCAs and 8 analysis years, 21,776 facility-year observations were available for the archetype assigning. Of these, 5,763 (26.5%) observations were assigned an archetype via nearest-neighbour imputation. About 62% of 5,763 imputations were attributed to years before 2021. At the HFCA level, 1,355 HFCAs (49.8%) were imputed in at least one year, of which 216 (7.9%) were imputed across the entire period.

Table S1. Overall proportion of HFCAs affected by nearest-neighbour imputation, 2017–2024.

Metric	Value
Facility-year observations (total)	21,776
Facility-year observations imputed	5,763 (26.5%)
Unique HFCAs (total)	2,722
HFCAs with at least one year imputation from 2017-2024.	1,355 (49.8%)
HFCAs imputed in all years (dropped in strict sensitivity)	216 (7.9%)

Non-randomness

Chi-square tests suggests an association between imputation status and province is medium-to-large by Cohen's benchmarks (Cramér's V = 0.38, $p < 0.001$); the associations with year (V = 0.17) and with archetype assignment (V = 0.15) are small in magnitude although also statistically significant given the large sample size.

Table S4. Chi-square tests of independence between imputation status and province, year, and archetype assignment.

Variable	Test	Statistic	df	p-value	Effect size
Imputation status vs province	χ^2 test	3,143.81	9	< 0.001	Cramér's V = 0.38

Variable	Test	Statistic	df	p-value	Effect size
Imputation status vs year	χ^2 test	619.66	7	< 0.001	Cramér's V = 0.17
Imputation status vs archetype	χ^2 test	460.51	3	< 0.001	Cramér's V = 0.15

Sensitivity analysis

To assess whether the non-random imputation pattern biases the archetype temporal stability and spatial clustering reported in the main manuscript, we recomputed the four stability statistics under three scenarios. The Baseline corresponds to the full dataset including all imputed assignments. The Lenient sensitivity drops only the imputed facility-year observations, retaining each HFCA for the years in which it has an original assignment. The Strict sensitivity drops any HFCA that has at least one imputed year over the analysis period, restricting the analysis to the 1,367 HFCAs with fully observed archetype assignments across all eight years.

Table S5. Archetype stability statistics under three imputation scenarios.

Scenario	n HFCAs / year-pair	Retention	Cohen's κ	ARI	Neighbour-sharing ratio
Baseline (full dataset)	2,722	0.84	0.77	0.61	2.86
Lenient (drop imputed facility-year observations)	1,857	0.86	0.80	0.65	2.58
Strict (drop HFCAs with ≥1 imputed year)	1,367	0.85	0.78	0.63	2.42

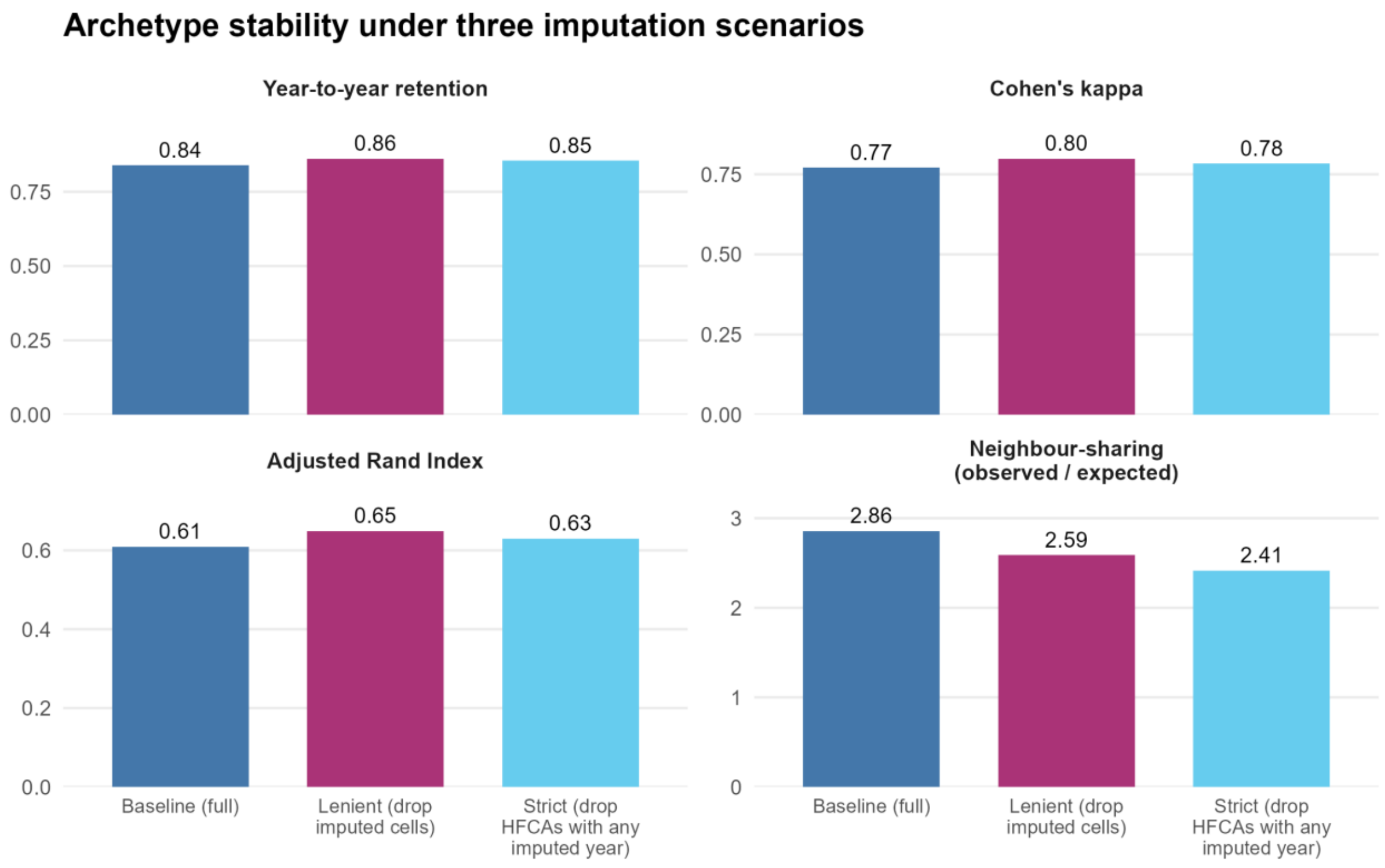

Figure S1. Archetype stability under three imputation scenarios. Year-to-year retention, Cohen's κ, Adjusted Rand Index, and the spatial neighbour-sharing ratio (observed share rate divided by expected by chance under random label permutation) are essentially unchanged across the Baseline, Lenient and Strict scenarios.

Conclusion

All four stability statistics are essentially unchanged across the three scenarios. Year-to-year retention ranges from 0.84 to 0.86, Cohen's κ from 0.77 to 0.80, and the Adjusted Rand Index from 0.61 to 0.65. The spatial neighbour-sharing ratio drops modestly from 2.86 (Baseline) to 2.42 (Strict). The permutation-based null distribution puts these ratios well outside the chance range under random archetype assignment ($p < 0.01$ across all three scenarios), so the identified archetypes remain strongly spatially clustered even under the most conservative subset. The year-to-year retention of approximately 86%, Cohen's κ = 0.79, Adjusted Rand Index = 0.64) correspond closely to the “Lenient” scenario.

We therefore conclude that the nearest-neighbour imputation procedure does not bias the temporal stability or spatial clustering of the identified archetypes. The non-random distribution of imputed HFCAs across provinces suggests underlying processes (catchment reorganisations over the years) rather than systematic data-quality bias against any province, and the conclusion that archetypes are temporally stable and spatially clustered holds under all three sensitivity scenarios.
